# Phosphoglycerate dehydrogenase interacts with and inhibits the protein kinase TAK1 to mitigate septic shock

**DOI:** 10.1016/j.jbc.2025.110658

**Published:** 2025-08-30

**Authors:** Penghui Hu, Zemin Ji, Hui Xiong, Sujun Yu, Xiao Shan, Hongyuan Dong, Weijia Jing, Jinrong Wang, Zhe Wang, Yan Cui, Baochen Wang, Yanzhao Zhou, Zihan Li, Jiuzhou Tang, Yan Cui, Ting Wang, Keliang Xie, Qiujing Yu

**Affiliations:** 1 Department of Critical Care Medicine, Tianjin Medical University General Hospital, Tianjin, China; 2 Tianjin Institute of Immunology, State Key Laboratory of Experimental Hematology, Key Laboratory of Immune Microenvironment and Disease (Ministry of Education), Department of Immunology, School of Basic Medical Sciences, Tianjin Medical University, Tianjin, China; 3 Department of Health Management Center & Institute of Health Management, Sichuan Provincial People's Hospital, School of Medicine, University of Electronic Science and Technology of China, Chengdu, China; 4 Department of Medical Oncology, The Affiliated Cancer Hospital of Zhengzhou University & Henan Cancer Hospital, Zhengzhou, China; 5 Department of Pathogen Biology, School of Basic Medical Sciences, Tianjin Medical University, Tianjin, China; 6 The Province and Ministry Co-sponsored Collaborative Innovation Center for Medical Epigenetics, School of Basic Medical Sciences, Department of Pharmacology and Tianjin Key Laboratory of Inflammation Biology, Tianjin Medical University, Tianjin, China; 7 Department of Anesthesiology, Tianjin Institute of Anesthesiology, Tianjin Medical University General Hospital, Tianjin, China

**Keywords:** PHGDH, macrophage, Sepsis, TAK1, LPS

## Abstract

Dysregulation of macrophage-mediated inflammatory responses is central to sepsis pathogenesis, making its modulation crucial for reducing organ damage and mortality. This study reveals that the key serine synthesis enzyme phosphoglycerate dehydrogenase (PHGDH), known for regulating tumor and immune cell functions, is significantly downregulated in mouse macrophages following lipopolysaccharide (LPS) stimulation, as well as in patients with systemic inflammatory response syndrome or sepsis. PHGDH knockdown enhances inflammatory responses to LPS and *Escherichia coli in vitro,* while myeloid PHGDH knockout exacerbates inflammation and organ damage in septic mouse models. In contrast, deficiency in serine and its derivative glycine inhibits LPS-induced macrophage inflammation both *in vitro* and *in vivo*. Mechanistically, PHGDH interacts with transforming growth factor-β–activated kinase 1 (TAK1), inhibiting TAK1 binding to TAK1-binding protein, thereby suppressing the TAK1–NF-κB/MAPK signaling pathway. Furthermore, adeno-associated virus–mediated PHGDH overexpression in lung macrophages reduces sepsis-related inflammation and damage, highlighting PHGDH's nonmetabolic role in regulating macrophage-mediated inflammation and suggesting new therapeutic strategies for sepsis treatment.

Imbalance in the inflammatory responses is a critical mechanism underlying sepsis, affecting its progression (1). Numerous studies have demonstrated that regulating the inflammatory responses is essential for mitigating organ damage in sepsis (2, 3). Macrophages (Mϕ) are innate immune cells characterized by high plasticity and heterogeneity, capable of differentiating into various subtypes in response to local microenvironmental cues, each performing distinct functions (4, 5). Under stimulation with the endotoxin lipopolysaccharide (LPS) from Gram-negative bacteria and/or interferon (IFN)-γ, resting M0 macrophages differentiate into classically activated M1 macrophages, which exhibit proinflammatory, bactericidal, and antitumor effects. Conversely, in response to interleukin (IL)-4 and IL-13, M0 macrophages can differentiate into alternatively activated M2 macrophages, which possess anti-inflammatory properties, promote tissue repair, and can support tumor growth (6). Therefore, elucidating the processes and mechanisms of M1 macrophage activation in sepsis is essential for understanding the pathogenesis of sepsis and developing treatment strategies.

Upon LPS stimulation, the Toll-like receptor 4 (TLR4) on macrophages recognizes LPS, forming a complex that initiates the inflammatory responses. During the recognition process of LPS, LPS first binds to LPS-binding protein to form a complex, which then interacts with the CD14 receptor on the cell membrane surface and is ultimately presented to the TLR4/MD-2 receptor complex. This binding process induces TLR4 dimerization and triggers conformational changes, thereby initiating downstream signal transduction. Depending on the signaling pathway, TLR4 primarily activates immune responses through two major signaling pathways: the MyD88-dependent pathway and the TRIF-dependent pathway. The former is rapidly activated *via* the membrane signaling platform of TLR4, driving proinflammatory responses, while the latter mediates delayed immune responses through the endosomal signaling platform, primarily characterized by IFN production.

In the MyD88-dependent signaling pathway, the TIR domain of TLR4 initially recruits the adaptor protein MyD88, which subsequently binds to IRAK4 and IRAK1 to form the Myddosome complex. The E3 ligase TRAF6 is then recruited to this TLR4 complex, where it binds to transforming growth factor-β–activated kinase 1 (TAK1) and catalyzes its polyubiquitination. This process promotes the formation of the TAK1–TAK1 binding proteins (TABs) complex, leading to the autophosphorylation and activation of TAK1 (7). Once activated, TAK1 phosphorylates downstream substrates such as NF-κB–inducing kinase and MAPK kinases MKK3/6 and MKK4, resulting in the activation of NF-κB, JNK, and p38 MAPK. JNK, in particular, plays a crucial role in the phosphorylation of c-Jun, a component of the AP-1 transcription factor complex, thereby facilitating its activation (8). These activated transcription factors, NF-κB and AP-1, translocate to the nucleus to induce inflammatory responses, including the expression of proinflammatory cytokines such as IL-6 and IL-1β, as well as chemokines like C-C motif chemokine ligand 5 (CCl5) (9, 10, 11, 12, 13). Additionally, TAK1 activation promotes the transcription of prosurvival genes, such as B-cell lymphoma 2 and inhibitor of apoptosis proteins, which protect cells from apoptosis induced by inflammatory stress, enhance their ability to adapt to stress, and subsequently promote cell survival and functional maintenance (14, 15, 16). These findings suggest that TAK1 may serve as a potential molecular target for inhibiting excessive inflammation and treating sepsis.

Serine is a nonessential amino acid that plays a critical role in various metabolic pathways, including glycolysis, one-carbon metabolism, lipid metabolism, and the synthesis of purines and glutathione (17). Cells can obtain serine from their environment *via* amino acid transporters or synthesize it *de novo* from glucose through the serine synthesis pathway (SSP), with the key initial reaction catalyzed by phosphoglycerate dehydrogenase (PHGDH). PHGDH governs the flux of glycolytic intermediates into the serine biosynthesis pathway, generating the majority of intracellular serine and its derivative glycine. These amino acids serve as precursors for nucleotides, glutathione, and one-carbon metabolism, positioning PHGDH as a central metabolic node in immune cells (18, 19, 20). Recent studies have demonstrated that serine metabolism enhances the LPS-induced expression of IL-1β in macrophages through mechanisms that include increased glutathione synthesis, or enhanced SAM-dependent H3K36 trimethylation (21, 22). Notably, serine deficiency has been shown to reduce proinflammatory cytokine levels in an LPS-mediated sepsis model in mice, alleviating LPS-induced inflammation (23). Additionally, myeloid-specific deletion of PHGDH or treatment with PHGDH inhibitors has been found to mitigate LPS-induced inflammation during sepsis (21, 22, 24). In contrast, our research indicates that PHGDH-mediated serine metabolism inhibits IFN-γ–activated M1 macrophage polarization *via* regulation of the SAM–IGF1–p38 axis (25). Furthermore, Wilson *et al.* found that PHGDH inhibition exacerbated inflammatory responses only when there was sufficient exogenous supply of serine and glycine (26). Therefore, it remains unclear whether the differential regulation of macrophage-mediated inflammatory responses by serine metabolism and PHGDH is linked to the nonmetabolic functions of PHGDH.

In this study, we demonstrate that PHGDH expression is significantly reduced in mouse macrophages following LPS stimulation and in patients with systemic inflammatory response syndrome (SIRS) or sepsis. Knockdown of PHGDH in macrophages enhances inflammation induced by LPS and *Escherichia coli* (*E. coli*). Furthermore, myeloid cell–specific deficiency of PHGDH exacerbates septic shock induced by LPS, *E. coli*, and cecal ligation and puncture (CLP) surgery. Mechanistically, PHGDH inhibits the interaction between TAB1 and TAK1 by directly binding to TAK1, a nonmetabolic function that suppresses TAK1 phosphorylation and downstream NF-κB and MAPK signaling pathways. Notably, overexpression of PHGDH in lung macrophages using adeno-associated virus (AAV) provides protection against LPS-induced septic shock. In summary, our findings position macrophage PHGDH as a potential therapeutic target for sepsis.

## Results

### PHGDH downregulates proinflammatory cytokine production in macrophages

**Figure 1:**
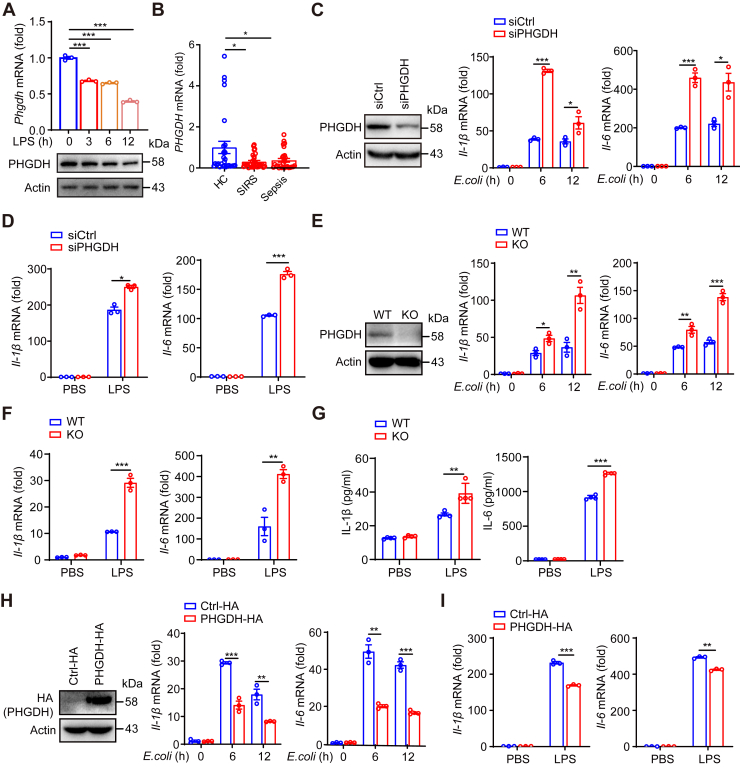
**PHGDH negatively regulates macrophage proinflammatory cytokines.***A*, PHGDH expression was measured by qPCR (*upper panel*) and Western blotting (*lower panel*) after stimulation of PMs with 500 ng/ml LPS for the indicated times. *B*, qPCR analysis of *PHGDH* mRNA expression in peripheral blood mononuclear cells (PBMCs) isolated from healthy human controls (HCs) and human patients with systemic inflammatory response syndrome (SIRS) or sepsis. *C* and *D*, RAW264.7 cells were transfected with siControl (siCtrl) or siPHGDH and then stimulated with *Esherichia coli* (1 × 10^6^ CFU) for indicated times (*C*) or 500 ng/ml LPS for 6 h (*D*). PHGDH protein expression was measured by Western blotting, and the mRNA expression levels of *Il-1β* and *Il-6* were measured by qPCR. *E*-*G*, peritoneal macrophages (PMs) (*E*) or bone marrow-derived macrophages (BMDMs) (*F* and *G*) from *Phgdh*^fl/fl^*Lyz2*-Cre^-^ (PHGDH-WT-Mϕ) and *Phgdh*^fl/fl^*Lyz2*-Cre^+^ (PHGDH-KO-Mϕ) mice were stimulated with 500 ng/ml LPS for 6 h or with *E. coli* (1 × 10^6^ CFU) for indicated times. Subsequently, Western blot analysis of PHGDH expression and qPCR (*E* and *F*) and ELISA analysis (*G*) of proinflammatory cytokine expression were conducted. *H* and *I*, RAW264.7 cells were transfected with control vector (Ctrl) or PHGDH-HA were stimulated with with *E. coli* (1 × 10^6^ CFU) for indicated times or 500 ng/ml LPS for 6 h. Subsequently, Western blot analysis was performed to assess HA (PHGDH) expression and qPCR analysis was conducted to evaluate the expression of the indicated genes. Data are presented as the means ± SEM. n = 3 per group (*upper panel of A, right panels of C, E, H, and D, F, I*); n = 4 per group (*G*); n = 28 to 30 per group (*B*). ∗*p* < 0.05, ∗∗*p* < 0.01, and ∗∗∗*p* < 0.001, two-tailed Student’s *t* test. CFU, colony-forming unit; IL, interleukin; LPS, lipopolysaccharide; PHGDH, phosphoglycerate dehydrogenase.

To investigate the role of macrophage PHGDH in LPS-induced inflammatory responses and the pathogenesis of sepsis, we first evaluated PHGDH expression in peritoneal macrophages (PMs) following LPS stimulation. Both mRNA and protein levels of PHGDH were significantly decreased after stimulation with and LPS (Fig. 1*A*). Additionally, the mRNA expression of PHGDH was significantly reduced in peripheral blood mononuclear cells (PBMCs) from patients with SIRS or sepsis (Fig. 1*B*).

We then found that depleting PHGDH using siPHGDH resulted in a significant increase in the expression of *Il-1β* and *Il-6* in *E. coli* or LPS-treated RAW264.7 cells (Fig. 1, *C* and *D*). Next, we utilized myeloid-specific PHGDH KO mice (*Phgdh*^fl/fl^*Lyz2*-Cre^+^, referred to as KO-Mϕ mice) and their *Phgdh*^fl/fl^*Lyz2*-Cre^-^ counterparts (WT-Mϕ mice), which we had previously generated (17, 25). In KO-Mϕ mice, the protein expression of PHGDH in splenic macrophages was significantly reduced, while there was no significant change in PHGDH expression in splenic NK cells, T cells, B cells, or dendritic cells (Fig. S1, *A*–*C*). We observed a similar upregulation of proinflammatory cytokines mRNA and protein levels in PMs and bone marrow–derived macrophages (BMDMs) with *Phgdh* ablation (Fig. 1, *E*–*G*). In contrast, overexpression of PHGDH led to a decrease in the expression of these proinflammatory cytokines induced by *E. coli*/LPS (Fig. 1, *H* and *I*). These results demonstrate that PHGDH suppresses the expression of proinflammatory cytokines and plays an anti-inflammatory role in macrophage-mediated inflammation.

Prolonged exposure to LPS can lead to a state of tolerance, characterized by a reprogramming of the inflammatory responses and a reduced production of inflammatory cytokines (27, 28). To investigate the role of PHGDH in the development of endotoxin tolerance following initial LPS activation, we first stimulated macrophages with low levels of LPS, followed by exposure to high levels of LPS to evaluate the expression of proinflammatory genes. Our findings indicated that both PHGDH deficiency and treatment with the PHGDH inhibitor CBR-5884 resulted in the induction of LPS tolerance in macrophages, with induction levels comparable to those in control cells (Fig. S1, *D* and *E*). This suggests that PHGDH does not play a significant role in macrophage endotoxin tolerance.

### Myeloid-specific PHGDH deficiency exacerbates LPS-induced septic shock

**Figure 2:**
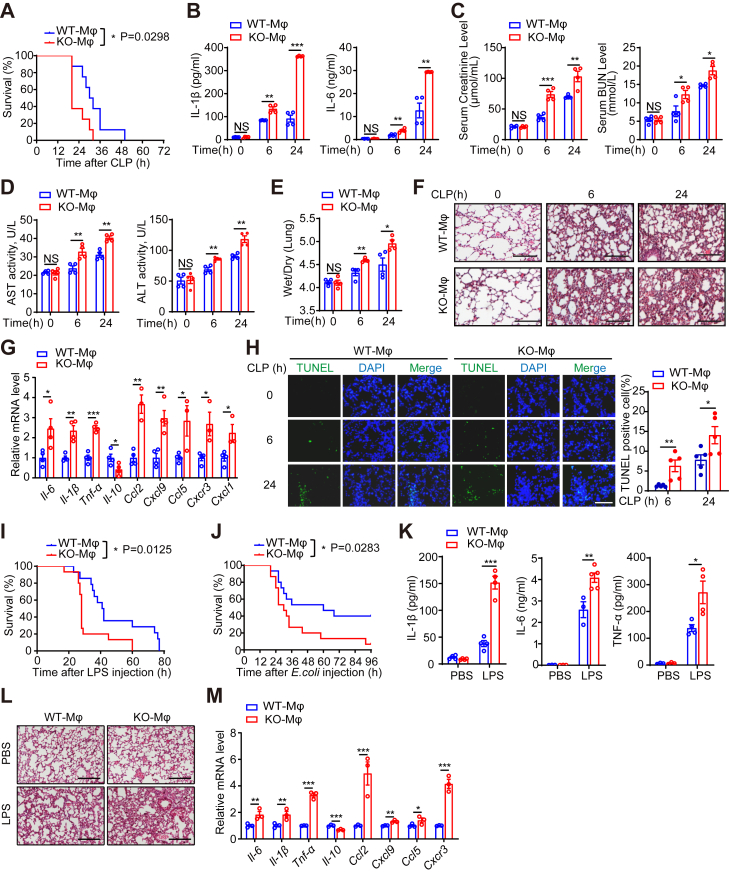
**Myeloid-specific PHGDH deficiency exacerbates LPS-induced septic shock.***A*–*M*, PHGDH-WT-Mϕ and PHGDH-KO-Mϕ mice were subjected to cecal ligation and puncture (CLP) surgery (*A*–*H*), or intraperitoneally injected with LPS (25 mg/kg) (*I*, *K*–*M*) or *Escherichia coli* (1 × 10^6^ CFU) dose (*J*). Survival rates of PHGDH-WT-Mϕ and PHGDH-KO-Mϕ mice subjected to CLP surgery (*A*). ELISA detection of serum IL-1β, IL-6 or TNF-α was performed at the indicated time points (*B*) or at 6 h after LPS challenge (*K*). ELISA detection of serum creatinine and blood urea nitrogen (BUN) levels (*C*), and serum aspartate aminotransferase (AST) and alanine aminotransferase (ALT) (*D*). The wet/dry weight ratios of the lung tissues were evaluated (*E*). Representative images of H&E staining of lung sections from the mice in the indicated groups are shown. Scale bars represent 100 μm (*F* and *L*). qPCR analysis was conducted to measure the levels of inflammation-related cytokines and chemokines in the lungs (*G* and *M*). The apoptosis assay was performed with TUNEL on lung tissue collected from PHGDH-WT-Mϕ and PHGDH-KO-Mϕ mice after CLP. The TUNEL signal is shown in fluorescent green and the stained nuclei are shown in *fluorescent blue* (DAPI). Represents the quantitative analysis result of the TUNEL stain signal. Scale bars represent 100 μm (*H*). Survival rates of PHGDH-WT-Mϕ and PHGDH-KO-Mϕ mice intraperitoneally injected with LPS (*I*) or *E. coli* (*J*). Data are presented as mean ± SEM. n = 8 per group (*A*); n = 3 to 5 per group (*B*-*E*, *G*, *H*, *K*, and *M*). n = 12 to 15 per group (*I* and *J*). ∗*p* < 0.05, ∗∗*p* < 0.01, and ∗∗∗*p* < 0.001, log-rank (Mantel-Cox) test (*A*, *I*, and *J*), or two-tailed Student’s *t* test, *B*–*E*, *G*, *H*, *K*, and *M*. CFU, colony-forming unit; IL, interleukink; LPS, lipopolysaccharide; PHGDH, phosphoglycerate dehydrogenase.

We further examined the impact of PHGDH deficiency on septic shock *in vivo* by challenging PHGDH-KO-Mϕ mice and their PHGDH-WT-Mϕ littermates with CLP surgery. Notably, KO-Mϕ mice exhibited lower survival rates after the CLP challenge (Fig. 2*A*). KO-Mϕ mice exhibited elevated levels of serum proinflammatory cytokines at both 6 and 24 h post-CLP challenge (Fig. 2*B*). Furthermore, KO-Mϕ mice demonstrated more severe kidney and liver injury compared to WT mice, as indicated by increased serum creatinine and blood urea nitrogen levels (Fig. 2*C*) and higher serum concentrations of aspartate aminotransferase (AST) and alanine aminotransferase (ALT) (Fig. 2*D*). Additionally, KO-Mϕ mice exhibited more pronounced significant lung injury, evidenced by a higher lung wet/dry weight ratio (Fig. 2*E*), as well as increased inflammatory cell infiltration and lung tissue damage, as revealed by H&E staining (Fig. 2*F*). They also showed increased expression of proinflammatory cytokines (*Il-6, Il-1β, and Tnf-α*), chemokines (*Ccl2, Cxcl9, Ccl5, and Cxcl1*), and *Cxcl3* receptor (*Cxcr3*), alongside a decrease in the production of the anti-inflammatory cytokine *Il-10* in their lungs (Fig. 2*G*). Furthermore, the percentage of apoptotic cells significantly increased in the lungs, as illustrated by the TUNEL assay (Fig. 2*H*).

Similarly, KO-Mϕ mice exhibited reduced survival rates after intraperitoneal injection of LPS or *E. coli* (Fig. 2, *I* and *J*). Furthermore, KO-Mϕ mice displayed more severe systemic and lung inflammation compared to WT mice after intraperitoneal injection of LPS (Fig. 2, *K*–*M*). These results indicate that myeloid-specific PHGDH deficiency exacerbates septic shock.

To confirm that PHGDH modulates other chemical-induced inflammatory responses, we assessed its role in a CCl_4_-induced acute hepatitis model. Forty-eight hours after the CCl_4_ challenge, PHGDH myeloid-deficient mice displayed more severe liver injury and inflammation, as evidenced by elevated serum levels of AST, and ratio of AST/ALT, as well as histological deterioration and altered expression of inflammation-related markers (Fig. S2, *A*–*C*). Next, we examined macrophage infiltration in liver sections through immunohistochemical staining. Our results showed that PHGDH myeloid deficiency significantly increased the number of IL-1β and IL-6 positive macrophages (Fig. S2*D*), further suggesting that PHGDH acts as a negative regulator of inflammatory responses.

### Deficiency in serine metabolism reduces macrophage-mediated inflammation and alleviates septic shock, in contrast to the effects of PHGDH deficiency

Considering that PHGDH is essential for the *de novo* synthesis of serine, we next investigated whether exogenous serine also has a similar anti-inflammatory effect. Our findings revealed that the deficiency of serine/glycine (SG) in BMDMs leads to a reduction in the expression of *Il-1β* and *Il-6* mRNA, while *Tnf-α* expression remains unaffected (Fig. S3*A*), consistent with previous studies (21).

To further explore the impact of serine restriction on sepsis *in vivo*, we maintained C57BL/6 mice on control or SG-free diets for 2 weeks, followed by intraperitoneal LPS injection. Mice subjected to serine restriction exhibited significantly lower serum serine concentrations (Fig. S3*B*), confirming the effectiveness of the dietary intervention. Additionally, these serine-restricted mice showed markedly decreased serum IL-1β and IL-6 levels (Fig. S3*C*), reduced lung injury (Fig. S3*D*), diminished production of proinflammatory cytokines and chemokines in the lungs (Fig. S3*E*). Overall, these findings suggest that SG deficiency suppresses macrophage inflammation and mitigates LPS-induced septic shock, contrasting with the effects observed with PHGDH deficiency. This indicates that PHGDH may influence macrophage-mediated inflammation through mechanisms that extend beyond its enzymatic role in serine synthesis.

### PHGDH suppresses LPS-mediated TAK1–NF-κB/MAPK signaling pathway

**Figure 3:**
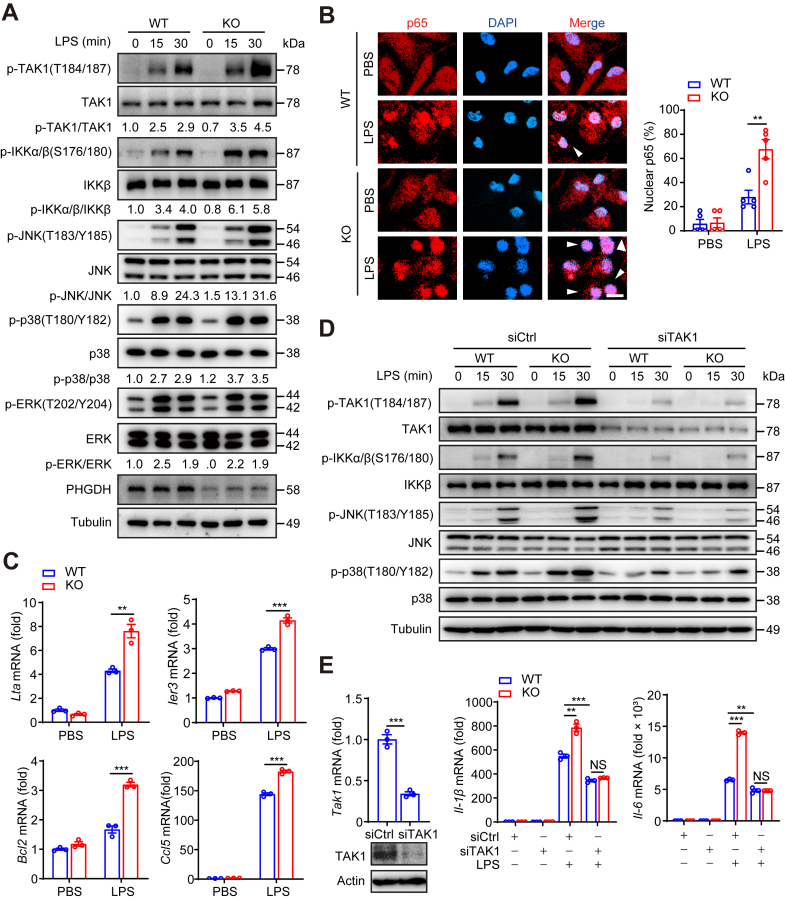
**PHGDH suppresses LPS-mediated TAK1–NF-κB/MAPK signaling pathway.***A*, Western blot analysis with the indicated antibodies. WT and KO BMDMs were treated with LPS for the indicated times. *B*, immunofluorescence staining was performed with anti-p65 antibody. WT and KO PMs were treated with 500 ng/ml LPS for 30 min. Representative images are shown. The scale bar represents 20 μm. The percentage of p65 localized in the nucleus was quantified by analyzing at least 50 cells across five randomly selected fields. *C*, PHGDH-WT and PHGDH-KO BMDMs were stimulated with 500 ng/ml LPS for 6 h, prior to qPCR analysis of NF-κB target gene expression. *D* and *E*, PHGDH-WT and PHGDH-KO BMDMs were transfected with siScramble (Scr) or siTak1 for 48 h and then stimulated with LPS for 30 min or 6 h, prior to Western blot analysis with the indicated antibodies p-TAK1(T184/187), TAK1, p-IKKα/β (S176/180), IKKβ, p-JNK (T183/Y185), JNK, p-p38 (T180/Y182), p38, PHGDH or tubulin (*D*) or qPCR analysis of proinflammatory cytokine expression (*E*). The data are presented as the means ± SEM. *n* = 3 to 5 per group (*B*, *C*, and *E*). ∗*p* < 0.05, ∗∗*p* < 0.01, and ∗∗∗*p* < 0.001; two-tailed Student’s *t* test. In (*B*) and (*C*), statistical analyses were performed with two-tailed Student’s *t* test. In (*E*), statistical analyses were performed with one-way ANOVA. BMDM, bone marrow–derived macrophage; LPS, lipopolysaccharide; PHGDH, phosphoglycerate dehydrogenase; PM, peritoneal macrophage; TAK1, transforming growth factor-β–activated kinase 1.

To investigate the potential regulatory mechanism of PHGDH in sepsis, we examined its impact on LPS-TLR4–mediated inflammatory signaling pathways. We treated BMDMs with LPS for various durations up to 120 min and found that p-TAK1 levels peaked at 30 min post-LPS stimulation in both WT and KO cells, followed by a significant decline after 60 min (Fig. S4*A*). Additionally, increased activation of IKKα/β, JNK, and p38 (but not ERK) was observed in PHGDH-KO BMDMs following 30 min of LPS stimulation (Fig. 3*A*). Immunofluorescence results indicated that PHGDH deficiency enhanced p65 nuclear translocation and the expression of NF-κB target genes, such as *Lta*, *Ier3*, *Bcl2*, and *Ccl5*, in response to LPS (Fig. 3, *B* and *C*).

Previous studies have established that TAK1 acts as a key upstream regulator converging on the NF-κB and JNK/p38 signaling pathways, leading to the expression of proinflammatory factors (9, 10, 29). We hypothesize that PHGDH may inhibit macrophage inflammatory responses by modulating TAK1 activity. Notably, the knockdown of TAK1 using siRNA or treatment with the TAK1 inhibitor 5Z-7-oxozeaenol (5Z-7-ox) reversed the activation of the TAK1–NF-kB/MAPK signaling pathway (Figs. 3*D* and S4*B*) and reduced the increased expression of proinflammatory cytokines (Fig. 3*E*) caused by PHGDH deficiency after LPS treatment.

Since TAK1 inhibition has been shown to induce macrophage death under both basal and LPS-treated condition (30, 31), we utilized the cell counting kit-8 (CCK-8) assay to evaluate the impact of TAK1 deficiency on the viability of PHGDH-WT and PHGDH-KO BMDMs. Pretreatment with 5Z-7-ox for 2 h, followed by 30 min of LPS treatment, did not significantly impact cell viability in either WT or KO cells (Fig. S4*C*). However, after a 2-h pretreatment with 5Z-7-ox followed by a 2-h LPS treatment, both WT and KO cells exhibited a significant decrease in cell viability to approximately 25%, with no notable difference between the two groups (Fig. S4, *C* and *D*). Additionally, KO cells did not show a significant difference in cell viability compared to WT cells under LPS stimulation alone (Fig. S4*D*). Additionally, our results indicated that cell viability decreased to approximately 70% 48 h after siTAK1 transfection in WT cells (Fig. S4, *E* and *F*). However, no significant differences in cell viability were observed between the WT and KO groups in either the basal or 6-h LPS-treated conditions. Therefore, it seems that PHGDH may not attenuate macrophage inflammatory responses through the modulation of TAK1 inhibition–mediated cell death.

In summary, these results suggest that PHGDH suppresses LPS-mediated TAK1–NF-κB/MAPK signaling pathways, thereby inhibiting macrophage inflammatory responses through modulation of TAK1 activity.

### PHGDH interacts with TAK1 *via* the amino acid (286–533) domain

**Figure 4:**
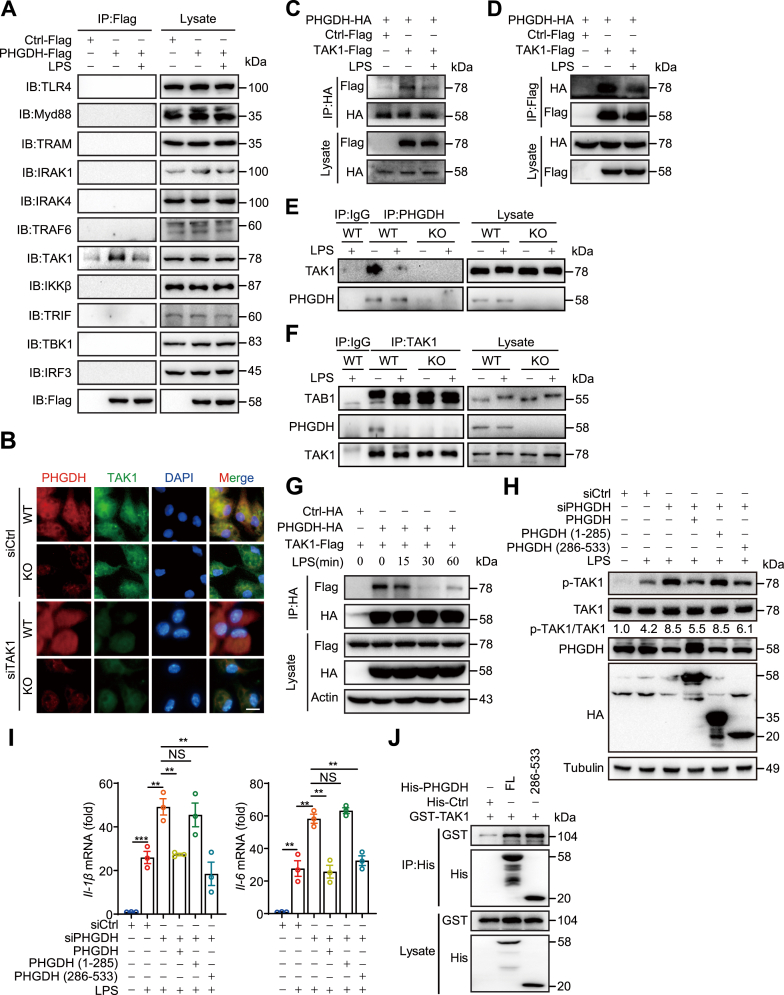
**PHGDH interacts with TAK1.***A*, immunoprecipitation was performed using RAW264.7 cell extracts with or without LPS stimulation. Exogenous PHGDH-Flag was immunoprecipitated with anti-Flag beads, while endogenous TAK1 was immunoprecipitated with an anti-TAK1 antibody. Immunoblot analysis was then conducted using the indicated antibodies. *B*, representative immunofluorescence staining images of TAK1 (*green*), and PHGDH (*red*) of PMs, in which nuclei were stained with DAPI (*blue*). Scale bars represent 10 μm. *C*–*F*, immunoprecipitation of exogenous PHGDH-HA with anti-HA-agarose (*C*) or TAK1-Flag with anti-Flag beads (*D*) or endogenous PHGDH with an anti-PHGDH antibody (*E*) or endogenous TAK1 with an anti-TAK1 antibody (*F*) and immunoblot analysis with the indicated antibodies were performed using TLR4-293T (*C* and *D*) or PM (*E* and *F*) extracts without or with LPS stimulation for 30 min. *G*, Ctrl-HA or PHGDH-HA and TAK1-Flag were expressed in TLR4-293T cells stimulated with LPS for the indicated times. Immunoprecipitation of exogenous PHGDH-HA with anti-HA-agarose and immunoblot analysis with the indicated antibodies were performed. *H*, protein levels of p-TAK1 (T184/187), TAK1, PHGDH, and HA in RAW264.7 cells transfected with siCtrl or siPHGDH and/or with the HA-Ctrl, HA-PHGDH, HA-PHGDH aa 1 to 285, or HA-PHGDH aa 285 to 533 plasmid and stimulated with LPS for 30 min were analyzed. *I* The expression levels of the proinflammatory cytokines *Il-1β* and *Il-6* were measured by qPCR in RAW264.7 cells transfected with siCtrl or siPHGDH and/or with the HA-Ctrl, HA-PHGDH, HA-PHGDH aa 1 to 285, or HA-PHGDH aa 285 to 533 plasmid and subsequently stimulated with LPS for 6 h. *J*, His pull-down assay results show the direct binding domains of PHGDH and TAK1. Purified GST/His were used as controls. The data are from three independent experiments performed with biological duplicates and are shown as the mean ± SEM (*n* = 3) (*I*) or are representative of three independent experiments (*A*, *C*–*H*, and *J*). NS, not significant (*p* ≥ 0.05), ∗∗*p* < 0.01 and ∗∗∗*p* < 0.001; one-way ANOVA. IL, interleukin; LPS, lipopolysaccharide; PHGDH, phosphoglycerate dehydrogenase; PM, peritoneal macrophage; TAK1, transforming growth factor-β–activated kinase 1; TLR4, Toll-like receptor 4.

To further investigate how PHGDH influences macrophage-mediated inflammatory signaling through TAK1, we overexpressed Flag-tagged PHGDH in RAW264.7 cells and performed coimmunoprecipitation assays to assess the interaction between key proteins in the TLR4 pathway and PHGDH. The results demonstrated that PHGDH interacts exclusively with TAK1 and does not interact with TLR4, MyD88, TRAM, IRAK1, IRAK4, TRAF6, IKKβ, TRIF, TBK1, and IRF3 (Fig. 4*A*). Immunofluorescence staining confirmed that PHGDH colocalizes with TAK1 within cells, and this colocalization is weakened in cells with PHGDH knockout and TAK1 knockdown (Fig. 4*B*). Additional bidirectional endogenous and exogenous coimmunoprecipitation experiments validated the interaction between the two proteins under both basal and LPS-stimulated conditions (Fig. 4, *C*–*F*). Time course analysis revealed a significant decrease in the interaction between PHGDH and TAK1 15 to 60 min after LPS treatment (Fig. 4*G*).

Next, we examined the specific PHGDH domains involved in the interaction with TAK1. PHGDH comprises two substrate-binding domains (aa 9–111 and aa 112–285), a nucleotide-binding domain (aa 286–317), and a regulatory domain (aa 322–533) (32). Overexpression of either WT PHGDH or the PHGDH (286–533) fragment, but not PHGDH (1–285) fragment, reversed the activation of the TAK1–NF-κB/MAPK signaling pathway and the increased expression of inflammatory factors caused by PHGDH deficiency under LPS stimulation (Fig. 4, *H* and *I*). This indicates that the aa 286 to 533 domain of PHGDH is crucial for its suppression of TAK1 activity. Consistent with this, purified recombinant PHGDH and PHGDH (286–533) were shown to interact with WT TAK1 (Fig. 4*J*). These results suggest that the aa 286 to 533 domain of PHGDH interacts with TAK1 to play a pivotal role in regulating macrophage inflammatory responses.

### PHGDH interacts with TAK1 in a tetrameric form

**Figure 5:**
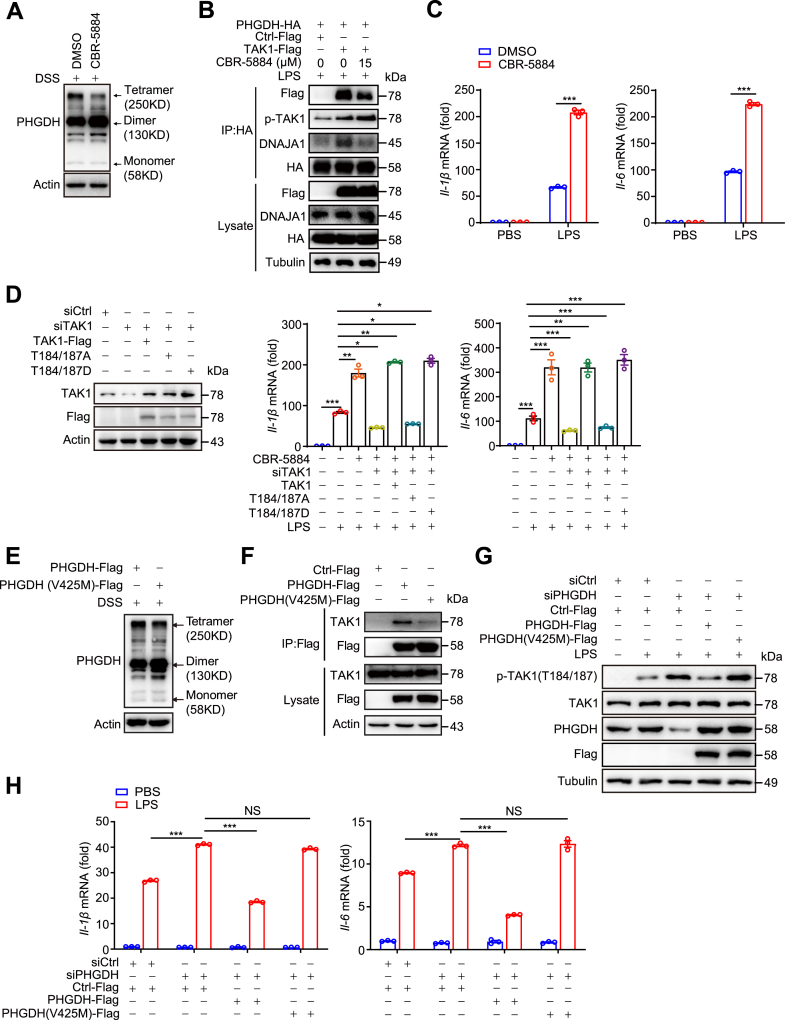
**PHGDH interacts with TAK1 in a tetrameric form.***A*, crosslinking with 0.25 mM disuccinimidyl suberate (DSS) and Western blot analysis of PHGDH in PMs pretreated with 15 μM ethyl 5-(2-furoylamino)-3-methyl-4-thiocyanato-2-thiophenecarboxylate (CBR-5884) for 6 h. *B*, immunoprecipitation of exogenous PHGDH-HA with anti-HA-agarose and immunoblot analysis with the indicated antibodies were performed using TLR4-293T cells pretreated with 15 μM CBR-5884 for 6 h and treated with LPS for 30 min. *C*, PMs were pretreated with 15 μM CBR-5884 for 6 h and then stimulated with LPS for 6 h. The mRNA levels of the proinflammatory cytokines *Il-1β* and *Il-6* were measured by qPCR. *D*, TAK1 and Flag expressions were measured by Western blotting (*left panel*) in RAW264.7 cells after transfected with siCtrl or siTAK1 and/or with the Flag-Ctrl-Flag, TAK1-Flag, TAK1-T184/187A-Flag, or TAK1-T184/187D-Flag plasmid. The expression levels of the proinflammatory cytokines *Il-1β* and *Il-6* were measured by qPCR (*right panel*) in RAW264.7 cells transfected with siCtrl or siTAK1 and/or with the Flag-Ctrl-Flag, TAK1-Flag, TAK1-T184/187A-Flag, or TAK1-T184/187D-Flag plasmid, pretreated with 15 μM CBR-5884 for 6 h, and stimulated with LPS for 6 h. *E*, PHGDH-KO-PMs were transfected with PHGDH-Flag or PHGDH (V425M)-Flag for 48 h, crosslinking was performed with 0.25 mM DSS, and the PHGDH complexes were evaluated by Western blotting. *F*, immunoprecipitation of exogenous Flag-PHGDH with anti-Flag beads and immunoblot analysis with the indicated antibodies were performed using TLR4-293T cells transfected with the Ctrl-Flag, PHGDH-Flag, or PHGDH(V425M)-Flag plasmids. *G* and *H*, RAW264.7 cells were transfected with siCtrl or siPHGDH and/or with the Ctrl-Flag, PHGDH-Flag, or PHGDH(V425M)-Flag plasmids and were then stimulated with LPS. The phosphorylated and total TAK1 protein levels were measured by Western blotting (*G*), and proinflammatory cytokine mRNA expression levels were measured by qPCR (*H*). The data are from three independent experiments performed with biological duplicates and are shown as the mean ± SEM (*n* = 3) (*C*, *D*, and *H*) or are representative of three independent experiments (*A*, *B*, *E*, *F*, and *G*). ∗*p* < 0.05, ∗∗*p* < 0.01, and ∗∗∗*p* < 0.001; two-tailed Student’s *t* test. In (*C*), statistical analyses were performed with two-tailed Student’s *t* test. In (*D*) and (*H*), statistical analyses were performed with one-way ANOVA. IL, interleukin; LPS, lipopolysaccharide; PHGDH, phosphoglycerate dehydrogenase; PM, peritoneal macrophage; TAK1, transforming growth factor-β–activated kinase 1; TLR4, Toll-like receptor 4.

The full-length PHGDH is found in solution as a dynamic mixture of monomers, dimers, and tetramers (33). To explore whether changes in PHGDH oligomerization influence its interaction with TAK1, we treated cells with the PHGDH inhibitor CBR-5884 (33, 34), which reduces PHGDH tetramers and increases its dimers (Fig. 5*A*). Subsequent analysis of the PHGDH–TAK1 interaction revealed that CBR-5884 treatment diminished PHGDH-TAK1 binding and significantly weakened the interaction with the molecular chaperone DNAJA1, which has been reported to facilitate PHGDH tetramerization (35), while TAK1 activity increased (Fig. 5*B*). These findings suggest that CBR-5884 disrupts the PHGDH–TAK1 interaction by inhibiting the formation of PHGDH tetramers. Furthermore, quantitative polymerase chain reaction (qPCR) analysis indicated that CBR-5884 treatment enhanced the expression of *Il-1β* and *Il-6* in macrophages (Fig. 5*C*).

To further investigate whether PHGDH tetramers inhibit macrophage inflammatory responses *via* TAK1, we knocked down TAK1 in RAW264.7 cells and then overexpressed TAK1, TAK1 (T184/187A) (which cannot be phosphorylated or activated), and TAK1 (T184/187D) (which is constitutively active). After pretreatment with CBR-5884, we evaluated the expression of inflammatory cytokines induced by LPS stimulation. The results indicated that both TAK1 and TAK1 (T184/187D) were effective in reversing the decrease in *Il-1β* and *Il-6* expression observed with TAK1 knockdown (Fig. 5*D*). These findings suggest that PHGDH tetramers inhibit macrophage inflammatory responses through the modulation of TAK1 activity.

V425 serves as the enzymatic active site of PHGDH and is located within the aa 286 to 533 domain that interacts with TAK1. We subsequently examined whether V425 influences the oligomeric state of PHGDH, thereby affecting its interaction with TAK1. Notably, we discovered that the V425M mutation in PHGDH reduces the formation of PHGDH tetramers while promoting the formation of dimers (Fig. 5*E*). Compared to WT PHGDH, the PHGDH (V425M) mutant significantly inhibited its binding to TAK1 (Fig. 5*F*). Furthermore, in contrast to WT PHGDH, PHGDH (V425M) was unable to restore TAK1 activation or the increased expression of proinflammatory cytokines triggered by PHGDH knockdown following LPS stimulation in RAW264.7 cells (Fig. 5, *G* and *H*). These findings further highlight the critical role of the tetrameric form of PHGDH in regulating macrophage inflammatory responses through its interaction with and inhibition of TAK1.

### PHGDH regulates LPS-induced inflammation by suppressing the interaction between TAB1 and TAK1

**Figure 6:**
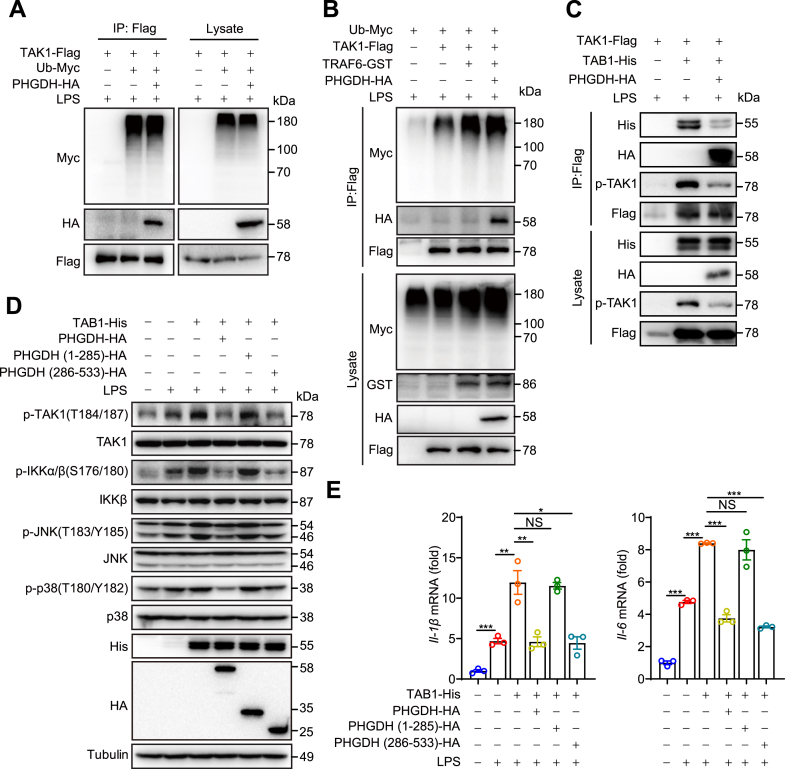
**PHGDH inhibits LPS-induced inflammation by suppressing the interaction between TAB1 and TAK1.***A* and *B*, the ubiquitination experiments showing the ubiquitination of exogenous TAK1 in TLR4-293T cells transfected with the indicated plasmids and treated with LPS for 30 min. *C*, immunoprecipitation assay results showing the interaction between TAB1 and TAK1 in RAW264.7 cells transfected with the indicated plasmids and treated with LPS for 30 min. *D*, protein levels of phosphorylated and total TAK1, IKKβ, JNK, p38, His, and HA in RAW264.7 cells transfected with the TAB1-His and/or, HA-PHGDH, HA-PHGDH aa 1 to 285, or HA-PHGDH aa 285 to 533 plasmids, as indicated, were measured after stimulation with LPS for 30 min. *E*, expression levels of the proinflammatory cytokines *Il-1β* and *Il-6* were measured by qPCR in RAW264.7 cells transfected with the TAB1-His and/or, HA-PHGDH, HA-PHGDH aa 1 to 285, or HA-PHGDH aa 285 to 533 plasmids as indicated, followed by LPS stimulation for 6 h. The data are from three independent experiments performed with biological duplicates and are shown as the mean ± SEMs (n = 3) (*E*) or are representative of three independent experiments (*A*-*D*). NS, not significant (*p* ≥ 0.05), ∗*p* < 0.05, ∗∗*p* < 0.01, and ∗∗∗*p* < 0.001; one-way ANOVA. LPS, lipopolysaccharide; IL, interleukin; PHGDH, phosphoglycerate dehydrogenase; PM, peritoneal macrophage; TAB1, TAK1-binding protein; TAK1, transforming growth factor-β–activated kinase 1; TLR4, Toll-like receptor 4.

To further investigate how the interactions between PHGDH and TAK1 regulate macrophage inflammatory responses, we employed coimmunoprecipitation to examine the effects of PHGDH on TAK1 ubiquitination and autophosphorylation. The results indicated that PHGDH did not significantly alter the overall level of TAK1 ubiquitination or TRAF6-mediated TAK1 ubiquitination (Fig. 6, *A* and *B*). However, PHGDH was found to inhibit the interaction between TAB1 and TAK1, leading to decreased levels of TAK1 phosphorylation (Fig. 6*C*). Moreover, overexpression of full-length PHGDH or the PHGDH (286–533) fragment, which contains the TAK1-binding domain, suppressed LPS-induced activation of the TAB1-mediated TAK1–NF-κB/MAPK signaling pathway and reduced proinflammatory cytokine expression. In contrast, the PHGDH (1–285) fragments were ineffective in this regard (Fig. 6, *D* and *E*). In summary, PHGDH regulates macrophage inflammatory responses by inhibiting the interaction between TAB1 and TAK1.

### AAV-mediated PHGDH overexpression in lung macrophages protects against LPS-induced septic shock

**Figure 7:**
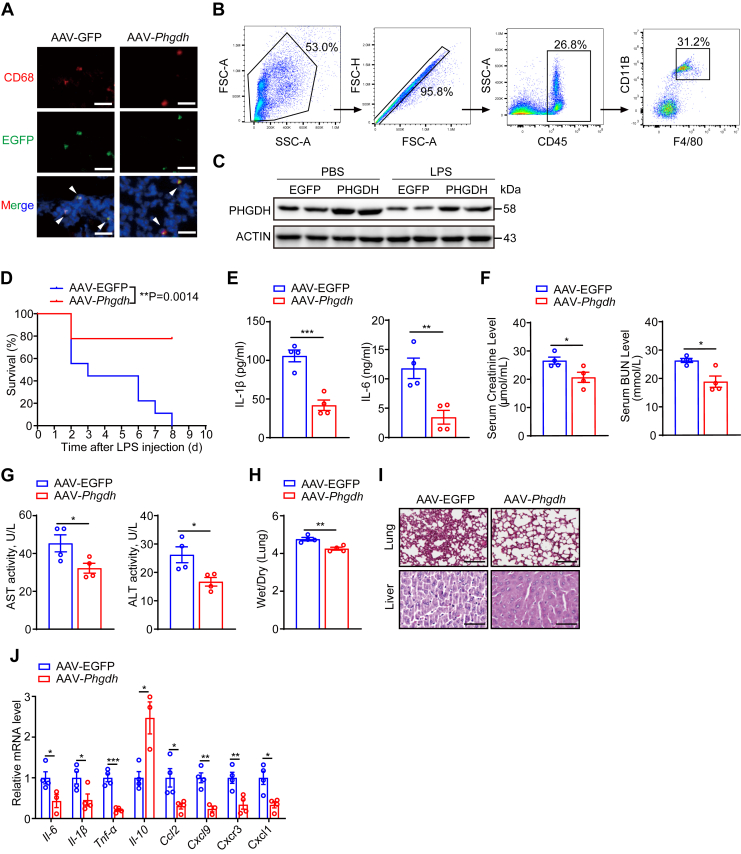
**Adeno-associated virus-mediated PHGDH overexpression in lung macrophages protects against LPS-induced septic shock.***A*, representative immunofluorescence images showing CD68 (*red*) and EGFP (*green*) staining in lung sections from mice in AAV-EGFP and AAV-*Phgdh* groups, with nuclei stained with DAPI (*blue*). Scale bars represent 50 μm. *B* and *C*, flow cytometry gating strategy for pulmonary macrophage (F4/80^+^CD11b^+^) (*B*). Western blot analysis of PHGDH expression in pulmonary macrophages of AAV-EGFP and AAV-*Phgdh* mice with PBS or LPS injection (*C*). *D*, survival rates of AAV-EGFP and AAV-*Phgdh* mice intraperitoneally injected with LPS (25 mg/kg). *E*, ELISA detection of serum IL-1β and IL-6. *F* and *G*, serum creatinine/urea (*F*) and AST/ALT (*G*) concentrations were measured in the mice in the indicated groups. *H*, the wet/dry weight ratios of the lung tissues were evaluated. *I*, representative images of lungs and liver sections from the mice in the indicated groups stained with H&E are shown. Scale bars represent 100 μm. *J*, qPCR analysis of inflammation-related cytokines and chemokines in lungs. Data are presented as mean ± SEM. n = 4 per group (*E*–*H* and *J*); n = 9 per group (*D*). ∗*p* < 0.05, ∗∗*p* < 0.01, and ∗∗∗*p* < 0.001, two-tailed Student’s *t* test, (*E*–*H*, and *J*), or log-rank (Mantel-Cox) test (*D*). ALT, alanine aminotransferase; AST, aspartate aminotransferase; IL, interleukin; LPS, lipopolysaccharide; PHGDH, phosphoglycerate dehydrogenase.

Finally, we assessed the therapeutic efficacy of lung macrophage PHGDH in LPS-induced sepsis model. To achieve this, we utilized a recombinant AAV serotype Lungx (AAV-Lungx) delivery system to overexpress *Phgdh* in mouse lung macrophages. Mice were injected *via* the tail vein with AAV-Lungx vectors carrying either mouse Phgdh (AAV-*Phgdh*) or an empty vector (AAV-EGFP) under the control of a CD68 promoter, which specifically targets macrophages. After a month, the mice were challenged with LPS. Immunofluorescence staining confirmed successful overexpression of PHGDH in the lung macrophages of AAV-*Phgdh*–injected mice (Fig. 7*A*). The immunofluorescence staining and Western blot results confirmed the overexpression of PHGDH in fluorescence-activated cell sorting (FACS)-sorted pulmonary macrophages of AAV-*Phgdh*–injected mice (Fig. 7, *A*–*C*). LPS treatment reduced endogenous PHGDH expression in the AAV-EGFP group compared to the untreated group. Notably, PHGDH expression was significantly higher in pulmonary macrophages from the AAV-*Phgdh* group compared to the AAV-EGFP group, both under untreated and LPS-stimulated conditions (Fig. 7*C*), suggesting that LPS has a minimal effect on the exogenous constitutive expression of PHGDH. Compared to the AAV-EGFP–injected controls, mice receiving AAV-*Phgdh* demonstrated improved survival rates (Fig. 7*D*). Furthermore, the AAV-*Phgdh* group exhibited significantly lower serum concentrations of proinflammatory cytokines (Fig. 7*E*), reduced damage to the kidneys, liver, and lungs, as well as decreased lung inflammation (Fig. 7, *F*–*J*).

## Discussion

PHGDH is the first rate-limiting enzyme in the *de novo* synthesis of serine. In this study, we observed a significant decrease in PHGDH expression in LPS-stimulated macrophages and in the PBMCs of patients with SIRS or sepsis (Fig. 1, *A* and *B*). Consistently, Wilson *et al.* demonstrated that LPS reduces PHGDH expression, while IL-4 increases it (26). They concluded that PHGDH expression and activity are characteristic of M2 macrophages. Conversely, recent studies have reported a significant increase in PHGDH expression in LPS-activated macrophages (22, 24). The underlying mechanisms driving these discrepancies warrant further investigation.

Recent research has demonstrated that metabolites derived from exogenous serine metabolism or PHGDH-mediated serine synthesis, such as glutathione and SAM, enhance proinflammatory cytokine expression in macrophages in response to LPS (21, 22). Additionally, a recent study revealed that PHGDH-mediated serine synthesis regulates sirtuin activity by competing for NAD^+^, thereby modulating the acetylation of histones and other proteins, which ultimately promotes IL-1β production in M1 macrophages, independent of serine metabolismderived metabolites (24). In our study, we found that a deficiency in exogenous serine metabolism inhibits inflammation in LPS-treated macrophages and in mouse models of sepsis, which is consistent with previous study. Conversely, depleting *Phgdh* in myeloid cells both *in vitro* and *in vivo* significantly exacerbates LPS-induced inflammation.

The differing outcomes among these studies can be attributed to several key factors: 1) Regulatory mechanisms: PHGDH may affect macrophage-mediated inflammation through mechanisms that extend beyond its role in serine metabolism, including nonmetabolic activities. Additionally, the deprivation of extracellular serine and glycine forces cells to rely solely on PHGDH-mediated SSP, which may induce metabolic stress. Although SSP flux may compensate, it might not fully offset limitations in substrates like 3-PG or NAD^+^, leading to metabolic disorders such as oxidative stress and ATP deficiency. This metabolic stress can activate stress signals (*e.g.*, ATF4, mTORC1), promoting the production of proinflammatory factors like IL-1β; 2) Nutritional variability: Differences in culture media composition or the availability of nutrients *in vivo* between studies may lead to opposing results. It is not yet fully clear whether the depletion of exogenous serine and its concentration in different culture medium affect the impact of PHGDH inhibition on the outcomes of inflammation-related experiments; 3) Methods of PHGDH inhibition: The SSP plays a crucial role in coordinating effective one-carbon flux beyond merely providing one-carbon units (17, 18, 22). Various chemicals and genetic techniques used to inhibit PHGDH may affect the extent to which one-carbon flux is suppressed and thus the outcomes of inflammation. For instance, CBR-5884 permanently inactivates PHGDH through covalent modification of Cys residue (Cys159), necessitating new enzyme synthesis for restoration (34). NCT-503 competitively inhibits NAD^+^ binding but can be reversed by high concentrations of NAD^+^ (20). PH-755 regulates dimer formation through allosteric binding, retaining 10 to 15% of basic activity (18, 36). Overall, these findings suggest that PHGDH may influence macrophage-mediated inflammation through mechanisms that extend beyond its role in serine synthesis and metabolism.

Our mechanistic study showed that PHGDH inhibits macrophage-mediated inflammation by interacts with TAK1, inhibiting its binding to TAB1, which suppresses the TAK1–NF-κB/MAPK signaling pathway and reduces LPS-induced proinflammatory cytokine expression. The potential nonmetabolic functions of PHGDH in influencing other macrophage and immune cell–mediated activities require further investigation. Furthermore, we found that PHGDH inhibitor CBR-5884 or PHGDH V425 enzymatic mutation, which affects its tetramer formation, inhibits the PHGDH–TAK1 interaction. This finding suggests caution in interpreting results from experiments where overexpression of active-site mutated metabolic enzymes fails to rescue phenotypes caused by gene knockout, as these mutations may also impact the enzyme's oligomeric state or interactions with other proteins. We also cannot rule out the possibility that PHGDH deficiency may influence mechanisms related to cytokine release, such as inflammasome activation.

TAK1 can be activated not only by LPS but also by cytokines such as transforming growth factor-β, IL-1β, and TNF-α, as well as by metabolic stress (37, 38, 39). It plays a critical role in various physiological and pathological processes, including cell growth, differentiation, survival, inflammatory diseases, and metabolic disorders (40). The activation of TAK1 is primarily regulated by posttranslational modifications (10, 41, 42, 43). Further investigations are necessary to assess whether PHGDH-mediated regulation of TAK1 posttranslational modifications and activity contributes to clinical diseases such as atherosclerosis, obesity, and diabetes.

Overall, PHGDH inhibits M1 macrophage polarization through its nonmetabolic functions. It achieves this by directly binding to TAK1 as a tetramer, preventing the interaction between TAB1 and TAK1 and thereby suppressing TAK1 phosphorylation and activation. Notably, overexpressing PHGDH in lung macrophages *via* AAV provides protection against LPS-induced septic shock. In summary, our findings indicate that macrophage PHGDH may be a promising therapeutic target for sepsis.

## Experimental procedures

### Mice

*Phgdh*^*fl/fl*^ mice, created *via* CRISPR-Cas9 gene editing, were obtained from Cyagen Biosciences. To generate myeloid cell-specific PHGDH KO mice and their corresponding littermates, *Phgdh*^fl/fl^ mice were crossbred with *Lyz2*-Cre mice provided by Dr Xiaoyue Tan (Nankai University). The genotypes of the mice were verified by PCR analysis of tail DNA using the following primer pairs. *Lyz2*-cre forward, 5′-CCCAGAAATGCCAGATTACG-3′; *Lyz2*-cre reverse, 5′-CTTGGGCTGCCAGAATTTCTC-3′. *Phgdh* lox forward, 5′-GCGTTTACAGCCATCTTCCTTCC-3′; and *Phgdh* lox reverse, 5′-ATCCTCTAAGCTGCATCCCTTATC-3′. All mice were maintained on a C57BL/6 background and housed in a specific pathogen-free facility under a 12-h light/12-h dark cycle at a constant temperature of 22 °C ± 2 °C. Animal care and experimental procedures were approved by the Ethics Committee of Tianjin Medical University.

### Cell lines and primary cell culture

TLR4-HEK293T, RAW264.7, and L929 cells were cultured in Dulbecco’s modified Eagle’s medium (DMEM) supplemented with 10% fetal bovine serum (FBS) and 1% penicillin-streptomycin. Mouse PMs were isolated using the Brewer thioglycolate medium injection method (Cat# 225650, BD). Each mouse received an intraperitoneal injection of 1 ml of the medium, and after 3 to 4 days, the mice were euthanized. Subsequently, 8 ml of cold PBS containing 3% FBS was injected into the abdominal cavity. The peritoneal fluid was then collected and centrifuged at 1400 rpm for 5 min. Finally, the cells were resuspended in DMEM for culture. To obtain BMDMs, we first isolated the tibia and femur from mice and then used a syringe to flush out the bone marrow and collect the cell suspension. After centrifugation, the cells were treated with red blood cell lysis buffer (Cat# R1010, Solarbio) at 4 °C for 10 min to lyse the red blood cells. Following the lysis treatment, the cells were centrifuged again and resuspended, and then cultured in a mixed medium of DMEM and L929 supernatant at a ratio of 2:1 for 7 days. In experiments investigating macrophage-mediated inflammation, macrophages were stimulated with *E. coli* (1 × 10^6^ colony-forming unit [CFU]) or LPS (Cat# L4516, Sigma-Aldrich) at specified concentrations and durations.

### Isolation of PBMCs

Human peripheral blood samples were obtained from patients in the ICU of Tianjin Medical University General Hospital. This study was approved by the Ethics Committee of Tianjin Medical University (IRB2024-YX-24301), and all procedures were performed in adherence to the Helsinki Declaration. All patients signed written informed consent to participate. Blood samples were collected by venipuncture, and PBMCs were separated by density gradient centrifugation using human peripheral blood lymphocyte separation medium (Cat# C0025, Beyotime) according to the manufacturer's instructions.

### CLP-induced sepsis model

Male PHGDH-WT-Mϕ and PHGDH-KO-Mϕ mice aged 6 to 8 weeks were anesthetized with isoflurane *via* intraperitoneal injection. Under sterile conditions, an abdominal incision was made, and the cecum was gently exteriorized. The cecum was then ligated at the distal end at either the half or three-fourths position. A 22-gauge needle was used to puncture between the ligation site and the distal end, allowing a small amount of feces to be squeezed out. The cecum was then returned to the abdominal cavity, and the abdominal wall was sutured. Following this, 37 °C saline (5 ml/100 g) was administered subcutaneously for resuscitation, along with analgesics and antibiotic treatment. In the sham-operated group, the cecum was removed without ligation or puncture. The survival time of mice was observed. Serum and tissue samples from the liver, kidneys, and lungs were collected at 6 h and 24 h for further analysis.

### LPS or *E. coli*–induced sepsis model

Male PHGDH-WT or PHGDH-KO mice aged 6 to 8 weeks were intraperitoneally injected with 25 mg/kg of LPS (Cat# L2880, Sigma-Aldrich) or *E. coli* (1 × 10^6^ CFU) to observe their survival time. After 6 h of LPS stimulation, the mice were sacrificed, and serum and lung tissue samples were collected for further analysis. To investigate the effect of serine on sepsis, C57BL/6 mice were maintained on either a control diet or a diet devoid of SG for 2 weeks, followed by intraperitoneal injection of LPS. To assess the therapeutic efficacy of lung macrophage PHGDH in LPS-induced sepsis model, we utilized a recombinant AAV serotype Lungx (AAV-Lungx) delivery system to overexpress *Phgdh* in mouse lung macrophages. Mice were injected *via* the tail vein with AAV-Lungx vectors carrying either mouse Phgdh (AAV-*Phgdh*) or an empty vector (AAV-EGFP) under the control of a CD68 promoter, which specifically targets macrophages. After a month, the mice were challenged with LPS.

### CCl_4_-induced acute hepatitis model

Mice were intraperitoneally injected with CCl_4_ (2.0 ml/kg; Cat# C27710, Acmec Biochemical) diluted in corn oil at a ratio of 1:3. After 48 h, the mice were sacrificed, and serum and liver samples were collected for further analysis.

### Assessment of lung wet/dry weight ratio

Following CLP treatment or LPS stimulation, the mice were euthanized, and the left lung tissue was removed for the measurement of wet weight. The wet lung sample was subsequently dried in an oven at 70 °C for 48 h to determine the dry weight. The wet/dry weight ratio was calculated by dividing the wet weight by the dry weight.

### Lung and liver histology

Lung or liver tissues from sepsis or acute liver injury models were fixed in paraformaldehyde, followed by paraffin embedding and sectioning. The lung or liver sections were stained with H&E, and then dehydrated and mounted. Finally, histological changes were observed under a light microscope (Olympus).

### Determination of serine concentration

The concentration of serine in serum was measured using the dl-serine assay kit (Cat# K743, BioVision). The experiments were conducted according to the manufacturer's instructions.

### Enzyme-linked immunosorbent assay

The levels of inflammatory factors IL-6 (Cat# EK0411), IL-1β (Cat# EK0394), and TNF-α (Cat# EK0527) in cell supernatants and serum were measured using the ELISA kits (Boster Biological Technology), and the experimental procedures were conducted according to the manufacturer's instructions.

### Kidney and liver injury

The commercial kits used were obtained from Nanjing Jiancheng Bioengineering Institute. Kidney injury was assessed by measuring serum creatinine (Cat# C011-2-1) and urea (Cat# C013-1-1) levels. Liver injury was evaluated by measuring serum AST (Cat# C010-2-1) and ALT (Cat# C09-2-1) levels. The experimental procedures were conducted according to the manufacturer's instructions.

### TUNEL assay

The TUNEL assay was performed using the One Step TUNEL Apoptosis Assay Kit (Cat# C1088, Beyotime) following the manufacturer's instructions. After the lung tissue sections were fixed and permeabilized, the TUNEL detection solution was applied, and the tissues were incubated at 37 °C for 1 h in the dark. The sections were then examined using a fluorescence microscope.

### Cell viability

The CCK-8 assay was employed to assess cell viability. PHGDH-WT and PHGDH-KO BMDMs were seeded in 24-well plates and transfected with either siCtrl or siTAK1 for durations of 0, 24, 48, and 72 h. At 48 h posttransfection, the cells were stimulated with 500 ng/ml LPS for 6 h. And cells were pretreated with 100 nM 5z-7-ox (Cat# O9890, Sigma-Aldrich) for 2 h, followed by 500 ng/ml LPS stimulation for 30 min. Then, 200 μl of medium and 20 μl of CCK-8 reagent (ApexBio Technology) were added to each well, and the cells were incubated at 37 °C for 1 to 4 h. The absorbance at 450 nm was subsequently measured using a microplate reader.

### Immunofluorescence staining

To assess the colocalization of F4/80 with IL-1β or IL-6, we utilized primary antibodies specific for F4/80 (1:200; Cat# ab16911, Abcam), IL-1β (1:200; Cat# 9722, Abcam), and IL-6 (1:200; Cat# AF-406-NA, R&D Systems) for immunofluorescence staining on 5-μm–thick frozen liver sections. Alexa Fluor 488– (Cat# R37120; Invitrogen) or Alexa Fluor 594–conjugated (Cat# R37117; Invitrogen) secondary antibodies were employed to visualize the primary antibodies. Nuclei in all images were counterstained with 4′,6-diamidino-2-phenylindole (Cat# S36939; Invitrogen), and images were captured using a fluorescence microscope. Furthermore, to investigate whether AAV-*Phgdh* targets macrophages in lung tissue, we conducted co-localization analysis of EGFP (Cat# ab-184601,Abcam) and CD68 (Cat# BA3638, Boster) on frozen lung sections.

To investigate the effect of PHGDH on the nuclear translocation of p65, PHGDH-WT and PHGDH-KO BMDMs were first seeded onto coverslips and stimulated with LPS for a specified time. The cells were then fixed and permeabilized, followed by staining with p65 antibody (Cat# 8242, Cell Signaling Technology). Next, a secondary antibody conjugated with Fluor 594 (Cat# R37117; Invitrogen) was used to detect the primary antibody. Images were acquired using a fluorescence microscope, and image analysis was performed using ImageJ software (https://imagej.nih.gov/ij/). To assess the colocalization of PHGDH with TAK1, PHGDH-WT, and PHGDH-KO PMs transfected with siCtrl or siTAK1 were stained with PHGDH antibody (Cat# ab240744, Abcam) and TAK1 antibody (Cat# sc-166562, Santa Cruz).

### Plasmid construction, plasmid transfection, and RNAi

Expression plasmids for PHGDH, TAK1, and TAB1 were constructed using standard molecular biology techniques and cloned into the pcDNA3.1-hemagglutinin (HA)/Flag, pLenti-3xFlag/HA, or pcDNA3-6xHisvector. The PHGDH and TAK1 mutant plasmids were constructed using site-directed mutagenesis, with plasmids encoding the WT PHGDH and TAK1 proteins as templates. Additionally, the plasmids with different structure domains of PHGDH (1–285 and 286–533) were generated based on pLenti-PHGDH-HA. The pCMV-Myc vector and ubiquitin-pCMV-Myc plasmids were provided by Dr Yupeng Chen (Tianjin Medical University), while the pcDNA5-glutathione-*S*-transferase (GST) vector and pcDNA5-TRAF6-GST plasmids were provided by Dr Hongliang Li (Wuhan University). RAW264.7, PMs, and TLR4-293T cells were transfected with the appropriate plasmids and siRNA using either Lipofectamine 2000 (Cat# 11668, Invitrogen) or Lipofectamine RNAiMAX (Cat# 3778, Invitrogen). The siRNA sequences were as follows: siPHGDH, 5′-GGGAGCGGGAGATCGAGAA-3′; siTAK1, 5′-AGAGAAGACAAACCATTATAA-3′.

### RNA extraction and RT-PCR

Total RNA was isolated from cells and tissues using TRIzol reagent (Cat# 15596018, Invitrogen) and subsequently reverse-transcribed into cDNA with a reverse transcription system (Cat# 24408, Bimake). Quantitative RT-PCR analysis was then conducted using SYBR Green PCR Mix (Cat# B21203, Bimake) along with specific primers in an ABI 7300 Detection System. *β-Actin* served as a housekeeping gene for normalization in the qPCR experiments. The qPCR primers used were as follows: *β-actin* forward, 5′-CGTACCACTGGCATCGTGAT-3′ and reverse, 5′-AGGTAGTCAGTCAGGTCCCG-3’; *Phgdh* forward, 5′-CCTCATTGTCCGGTCTGCTAC-3′ and reverse, 5′-CATCTTTCATCGAAGCTGTTGC-3′; *PHGDH* forward, 5′-CACGACAGGCTTGCTGAATGA-3′ and reverse, 5′-CACGACAGGCTTGCTGAATGA-3′; *Il-1 β* forward, 5′-GAAATGCCACCTTTTGACAGTG-3′ and reverse, 5′-TGGATGCTCTCATCAGGACAG-3′; *Il-6* forward, 5′-TTGCCTTCTTGGGACTGAT-3′ and reverse, 5′-TTGCCATTGCACAACTCTT-3′; *Tnf-α* forward, 5′-CCTCTTCTCATTCCTGCTTG-3′ and reverse, 5′-GTCACCCCGTCCACATCTT-3′; *Il-10* forward, 5′-CAACATACTGCTAACCGACTC-3′ and reverse, 5′-CATGGCCTTGTAGACACCT-3′; *Ccl2* forward, 5′-TTAAAAACCTGGATCGGAACCAA-3′ and reverse, 5′-GCATTAGCTTCAGATTTACGGGT-3′; *Cxcl9* forward, 5′-TCCTTTTGGGCATCATCTTCC-3′ and reverse, 5′-TTTGTAGTGGATCGTGCCTCG-3′; *Ccl5* forward, 5′-GCTGCTTTGCCTACCTCTCC-3′ and reverse, 5′-TCGAGTGACAAACACGACTGC-3′; *Cxcr3* forward, 5′-TACCTTGAGGTTAGTGAACGTCA-3′ and reverse, 5′-CGCTCTCGTTTTCCCCATAATC-3′; *Cxcl1* forward, 5′-CTGGGATTCACCTCAAGAACATC-3′ and reverse, 5′-CAGGGTCAAGGCAAGCCTC-3′; *Lta* forward, 5′-CCACCTCTTGAGGGTGCTTG-3′ and reverse, 5′-CATGTCGGAGAAAGGCACGAT 3′; *Ier3* forward, 5′-GCTCTGGTCCCGAGATTTTCA-3′ and reverse, 5′-AGATGATGGCGAACAGGAGAA-3′; *Bcl2* forward, 5′-GTCGCTACCGTCGTGACTTC-3′ and reverse, 5′-CAGACATGCACCTACCCAGC-3′; *Tak1* forward, 5′-CGGATGAGCCGTTACAGTATC-3′ and reverse, 5′-ACTCCAAGCGTTTAATAGTGTCG-3′.

### Protein isolation, immunoprecipitation, and Western blotting

For immunoblotting, cells were lysed using cold radioimmunoprecipitation assay lysis buffer containing a protease inhibitor cocktail. For immunoprecipitation, proteins from cultured cells were extracted with cold native lysis buffer (Cat# R0030, Solarbio) supplemented with a protease inhibitor cocktail and then incubated with Flag/HA agarose beads or antibodies against PHGDH (1:200, Cat# ab240744, Abcam)/TAK1 (1:200, Cat# 5206, CST). The expression and interaction of proteins were analyzed by Western blotting. After SDS-PAGE, proteins were transferred onto a polyvinylidene fluoride membrane (Bio-Rad), blocked with 5% milk, and probed with specific antibodies diluted in primary antibody dilution buffer (Beyotime), including PHGDH (1:1000, Cat# ab240744, Abcam); TAK1(1:1000, Cat# 5206, CST); TAK1(1:1000, Cat# CY7057, Abways); p-TAK1 (T184/187) (1:1000, Cat# 4508, CST); IKKβ (1:1000, Cat# 8943, CST); p-IKKα/β (S176/180) (1:1000, Cat# 2697, CST); JNK (1:1000, Cat# 9252T, CST); p-JNK (T183/Y185) (1:1000, Cat# 4668T, CST); p38 (1:1000, Cat# 8690T, CST); p-p38 (T180/Y182) (1:1000, Cat# 4511T, CST); ERK(1:2000, Cat# 4695T, CST); p-ERK (T202/Y204) (1:1000, Cat# 4370T, CST); p65 (1:1000, Cat# 8242, CST); HA (1:2000, Cat# 3724S, CST), Flag (1:2000, Cat# F1804, Sigma); His (1:1000, Cat# AH367, Beyotime); GST 1:1000, (1:1000, Cat# 10000-0-AP, Proteintech); Myc (1:1000, Cat# 2278S, CST); cleaved caspase1 (1:1000, Cat# BY9026, Abways); cleaved-caspase 3 p17 (1:1000, Cat# 341034, Zenbio); cleaved-caspase 8 (1:1000, Cat# 250106, Zenbio); actin (1:3000, Cat# sc-47778, Santa Cruz); and tubulin (1:3000, Cat# ab7792, Abcam). The membrane was then incubated with horseradish peroxidase-conjugated goat anti-rabbit IgG (1:5000, Cat# ZB-2301, ZSGB-BIO) or horseradish peroxidase-conjugated goat anti-mouse IgG secondary antibodies (1:5000, Cat# ZB-2305, ZSGB-BIO). Visualization was performed using an ECL chemiluminescence kit (Millipore) and a chemiluminescence imaging system (Tanon).

### Recombinant protein purification and His pulldown

Purified PHGDH or PHGDH-(286–533) was obtained from BL21 (DE3) competent *E. coli* cells transformed with plasmids containing the pet28a-His tag. Similarly, TAK1 was purified from BL21 (DE3) cells transformed with pGEX-4T-1-GST–tagged plasmids. The cultures were grown at 37 °C until the absorbance at 600 nm reached 0.6, followed by induction with 0.5 mM IPTG at 25 °C for 16 h. After harvesting, the bacterial pellets were lysed *via* sonication in a buffer containing 20 mM Tris (pH 7.5), 0.1% Triton X-100, 1 mg/ml lysozyme, and 1 mg/ml DNase. His-tagged PHGDH was purified using TALON Metal Affinity Resins (Cat# 635503, Takara) and eluted with 100 mM imidazole. The eluted proteins were then concentrated in a buffer of 25 mM Tris-Cl and 0.4 M NaCl (pH 8.0). GST-tagged TAK1 was enriched using GST-Sefinose resin (Cat# C600031, Sangon) and eluted with 10 mM reduced glutathione in 50 mM Tris (pH 8.0). The eluted proteins were dialyzed overnight against PBS prior to pull-down assays. The purified proteins were mixed and incubated as described in the figure, followed by pulldown using TALON Metal Affinity Resins. The resulting eluted proteins were analyzed *via* SDS-PAGE.

### Crosslinking

In WT-PM cells treated with 15 μM CBR-5884 for 6 h, or in KO-PM cells transfected with PHGDH-HA or PHGDH (V425M)-HA, native lysis buffer (Cat# R0030, Solarbio) was added, and the cells were lysed on ice for 10 min. The cells were then collected, and the supernatant was obtained by centrifugation at 12,000 rpm for 10 min. Next, 0.25 mM dimethyl suberimidate (Cat# 20700, Thermo Scientific Pierce) was added to the supernatant and incubated at room temperature for 10 min for crosslinking. Subsequently, 1 M Tris (pH 7.5) was added to achieve a final concentration of 20 mM, and the reaction was quenched at room temperature for 15 min. Finally, the lysate was analyzed by Western Blot.

### Flow cytometry

To further validate the efficiency of PHGDH under the *Lyz2*-cre *in vivo*, we isolated splenic immune subsets (CD11b^+^F4/80^+^ macrophages, NK1.1^+^ NK cells, CD4^+^ T cells, CD8^+^ T cells, CD19^+^ B cells, and CD11c^+^ MHCII^+^ dendritic cells) from both PHGDH-WT-Mϕ and PHGDH-KO-Mϕ mice by FACS. Spleen cells of mice were extracted by mechanical method. Specifically, the spleen of mice was placed in a 2 mL eppendorf tube containing 1 ml of cold 3% FBS. The spleen was carefully minced with surgical scissors. The minced spleen was passed through a 70 μm cell strainer and ground with the bottom of a disposable syringe. After centrifugation at 1400 rpm for 5 min, the supernatant was discarded and the cells were treated with red blood cell lysis buffer (Cat# R1010, Solarbio) at 4 °C for 10 min to lyse the red blood cells. After centrifugation, the cells were resuspended in PBS to prepare a single-cell suspension. For sorting, the cells were blocked with CD16/32 antibody (1:200, Cat# 156603, BioLegend) and incubate on ice in the dark for 5 to 10 min. Then, the cells were incubated with Zombie NIR Fixable Viability APC-Cy7 (1:1000, Cat# 423105, BioLegend) at room temperature for 15 to 30 min. And the cells were stained by fluorescence-conjugated antibodies of CD45 FITC (1:200, Cat# 03107, BioLegend), F4/80 PE-Cy7 (1:200, Cat# 123113, BioLegend), CD11b APC (1:200, Cat# 101211, BioLegend), NK1.1 PE (1:200, Cat# 108707, BioLegend), CD4 PerCp-Cy5.5 (1:200, Cat# 100433, BioLegend), CD8 PE-Cy7 (1:200, Cat# 100721, BioLegend), CD19 APC (1:200, Cat# 152409, BioLegend), CD11c APC (1:200, Cat# 117309, BioLegend), and MHC II PE (1:200, Cat# PE-65122, Proteintech) for 30 min on ice in the dark. Splenic immune subsets were sorted using a FACS Aria Fusion Cell Sorter (BD Biosciences), and data were analyzed using FlowJo software (https://www.flowjo.com/).

To sort pulmonary macrophages, lung tissues from AAV-EGFP or AAV-*Phgdh* mice injected with PBS or LPS was excised and processed into a single-cell suspension after digestion with collagenase IV (Cat# 17104019; Gibco) at 37 °C for 30 min. Pulmonary immune cells were then further purified from the single-cell suspension by density gradient centrifugation using 30% Percoll (Cat# P8370, Solarbio). The cell suspension was subsequently stained with fluorescence-conjugated antibodies: CD45 PE (1:100, Cat# 103105, BioLegend), F4/80 PE-Cy7 (1:100, Cat# 123113, BioLegend), and CD11b APC (1:100, Cat# 101211, BioLegend). Lung macrophages (CD45^+^F4/80^+^CD11b^+^) were sorted using a FACS Aria Fusion Cell Sorter.

### Statistical analyses

The figure legends provide details on the sample sizes and significant differences between the samples. The significance levels of two groups were determined by two-tailed Student’s *t* test. One-way analysis of variance was used to compare the data among multiple samples. Survival rate differences were evaluated with the log-rank (Mantel-Cox) test. Fluorescence imaging analyses were conducted in a blinded manner. All quantitative data are expressed as the mean ± SEM from at least three independent experiments. Statistical analyses were carried out using GraphPad Prism software (https://www.graphpad.com/), with a *p* value of <0.05 considered significant. Notably, NS indicates not significant (*p* ≥ 0.05); ∗*p* < 0.05; ∗∗*p* < 0.01; and ∗∗∗*p* < 0.001.

## Data availability

Derived raw data supporting the results of this study are available from the corresponding author on request.

## Supporting information

This article contains supporting information.

## Conflict of interests

The authors have declared that no competing interests exist.
