# Supplementary material for: Phosphoglycerate dehydrogenase interacts with and inhibits the protein kinase TAK1 to mitigate septic shock
Source: J Biol Chem. 2025 Aug 30;301(10):110658. doi: 10.1016/j.jbc.2025.110658 (PMC12509985; doi:10.1016/j.jbc.2025.110658)
Supplement: Supporting Figures [file mmc1.docx]

**Phosphoglycerate dehydrogenase interacts with and inhibits the protein kinase TAK1 to mitigate septic shock**

Penghui Hu, Zemin Ji, Hui Xiong, Sujun Yu, Xiao Shan, Hongyuan Dong, Weijia Jing, Jinrong Wang, Zhe Wang, Yan Cui, Baochen Wang, Yanzhao Zhou, Zihan Li, Jiuzhou Tang, Yan Cui, Ting Wang, Keliang Xie, Qiujing Yu

**This file includes:**

Fig. S1 PHGDH deficiency had no effect on LPS tolerance

Fig. S2 Myeloid-specific PHGDH deficiency exacerbates CCl_4_-induced acute liver injury

Fig. S3 Ser/Gly deficiency suppresses macrophage LPS-induced inflammation and restricts septic shock

Fig. S4 PHGDH suppresses LPS-mediated TAK1-NF-κB /MAPK signaling pathway

Fig. S5 Phosphoglycerate dehydrogenase interacts with and inhibits TAK1 activity to mitigate septic shock

**Figure S1**

**
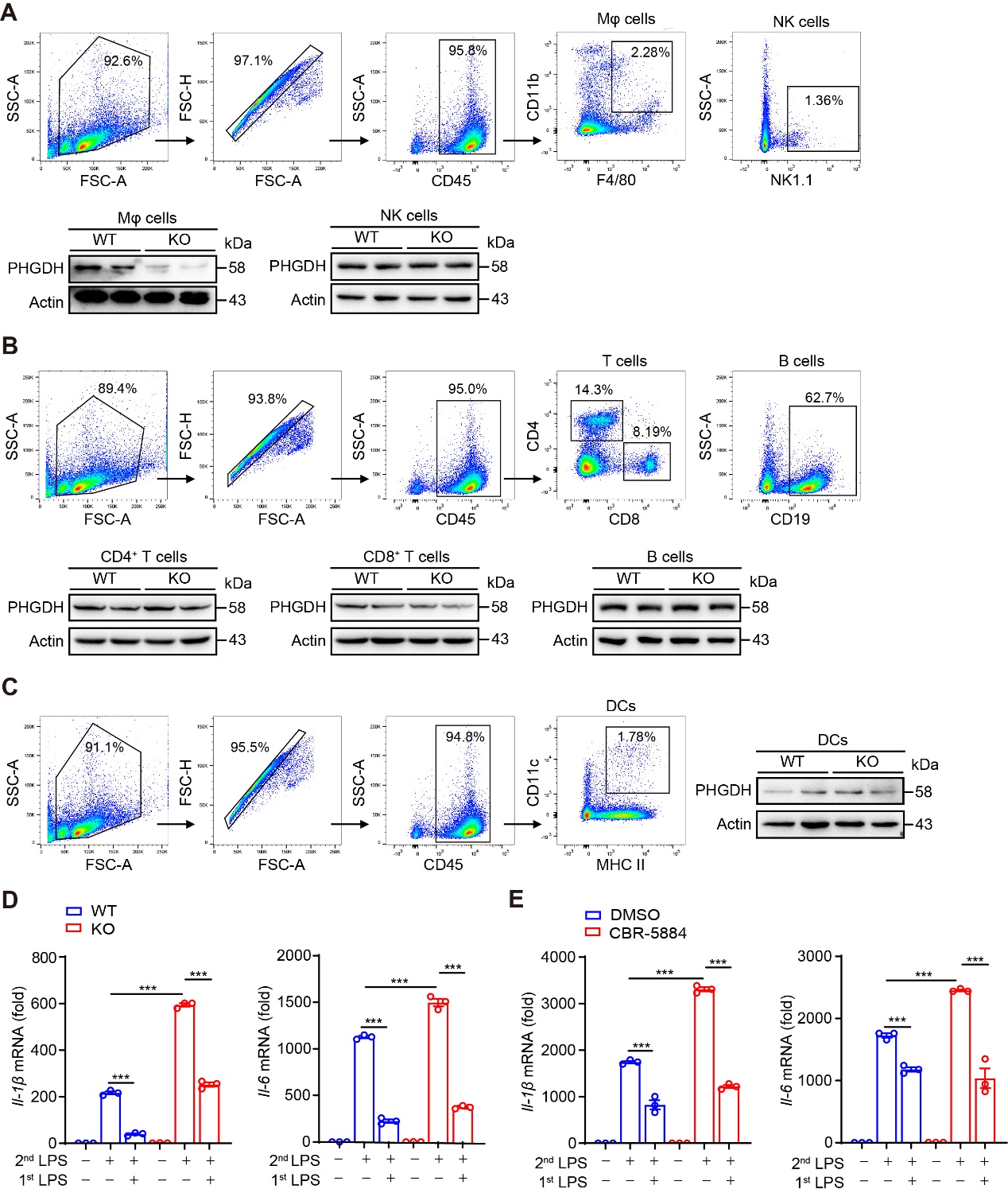
**

**Fig. S1** PHGDH deficiency had no effect on LPS tolerance

**A-C** Isolation and characterization of splenic immune subsets from PHGDH-WT-Mφ and PHGDH-KO-Mφ mice: flow cytometry gating strategy of macrophages, NK cells, T cells, B cells and DCs, and western blot analysis of PHGDH expression.

**D-E** WT and KO PMs **(D)**, as well as WT PMs pretreated with or without CBR-5884 for 6 h **(E)**, were pretreated with or without LPS (10 ng/mL) for 6 h, followed by stimulation with LPS (500 ng/mL) for 6 h. Subsequently, qPCR analysis of the indicated pro-inflammatory cytokines was performed.

Data are presented as the means ± SEM. n = 3 per group (D and E). ***p < 0.001, one-way ANOVA.

**Figure S2**


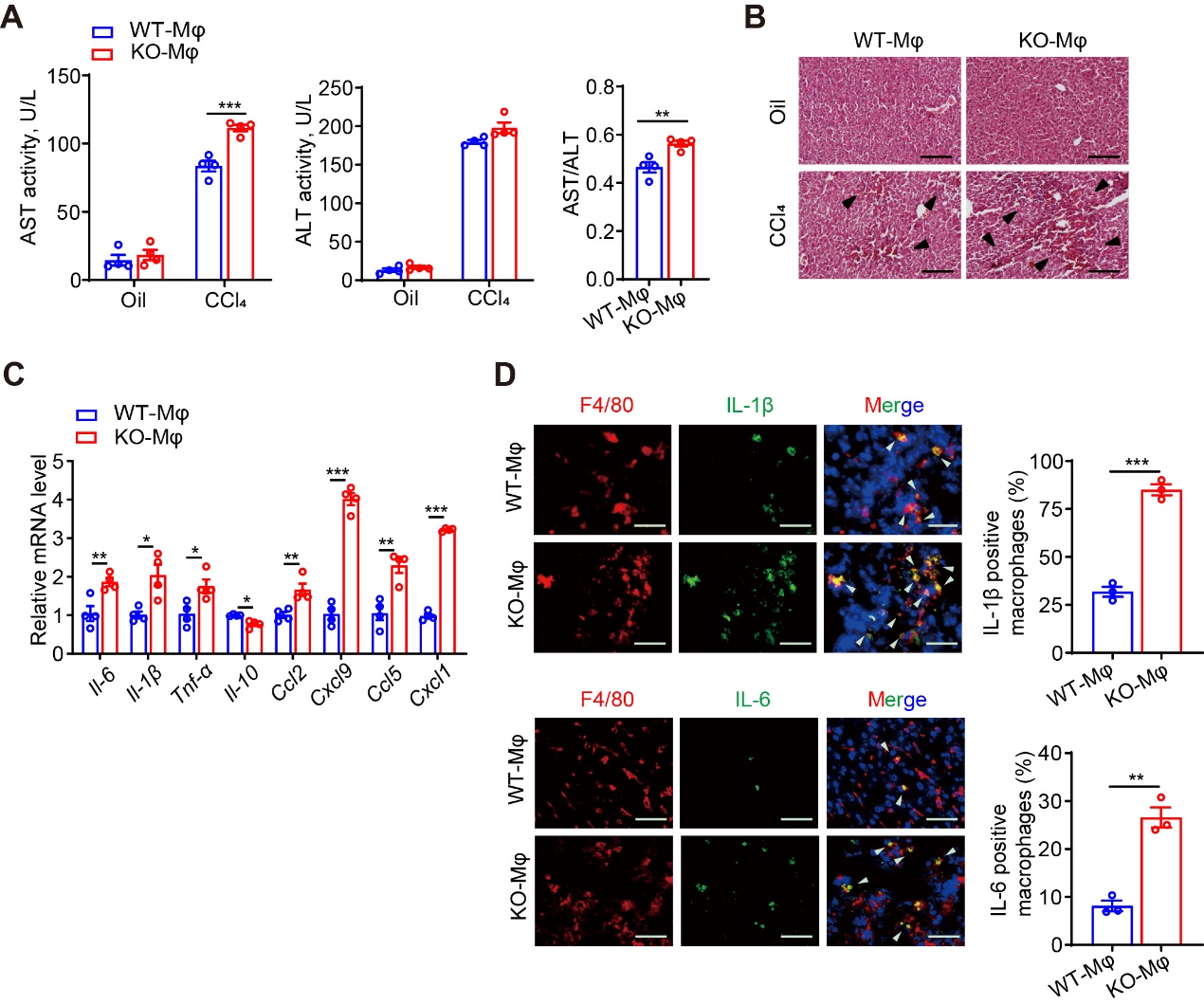


**Fig. S2** Myeloid-specific PHGDH deficiency exacerbates CCl_4_-induced acute liver injury

**A–D** Acute liver injury was induced in PHGDH-WT-Mφ and PHGDH-KO-Mφ mice by intraperitoneal injection of CCl_4_ for 48 h. The concentrations of serum aspartate aminotransferase (AST) and alanine aminotransferase (ALT) were determined **(A)**. Representative images of hematoxylin and eosin (H&E) staining of liver sections from the mice in the indicated groups are shown; scale bars, 100 μm (**B**). qPCR analysis of the indicated cytokines and chemokines was performed **(C)**. Liver sections of PHGDH-WT-Mφ and PHGDH-KO-Mφ mice were stained for immunofluorescence with the indicated antibodies, and representative images are shown. Scale bars, 50 μm. IL-6 and IL-1β-positive macrophages were quantified **(D)**. Data are presented as mean ± SEM. n = 3-4 per group (A, C and D). *p < 0.05, **p < 0.01, ***p < 0.001, two-tailed Student’s t test.

**Figure S3
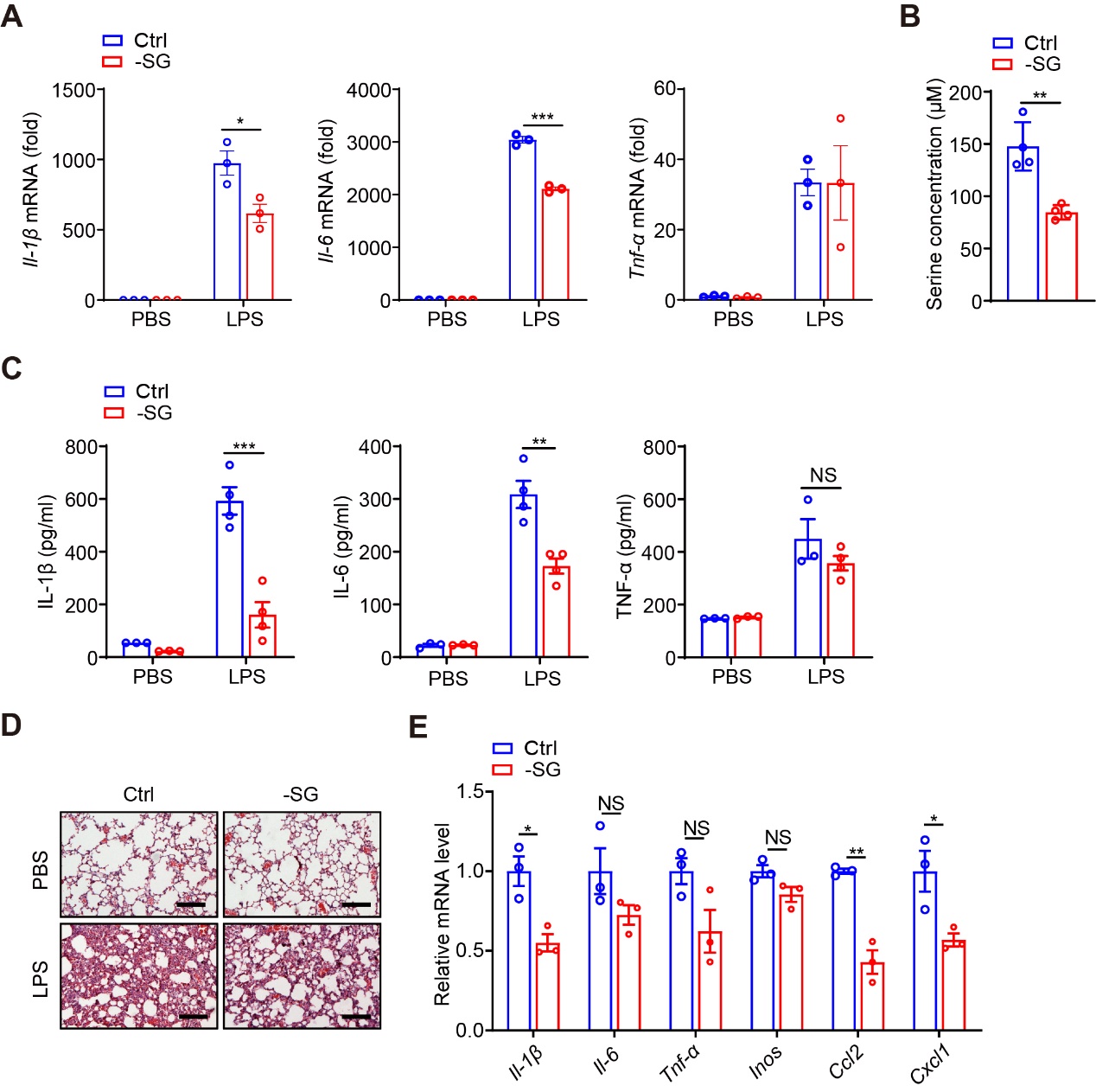
**

**Fig. S3** Ser/Gly deficiency suppresses macrophage LPS-induced inflammation and restricts septic shock

**A** BMDMs were starved of serine and glycine for 12 h, then stimulated with 500 μg/mL LPS for 6 h, and the mRNA expression levels of the proinflammatory cytokines *Il-6*, *Il-1β* and *Tnf-α* were measured by qPCR.

**B-E** C57BL/6 mice pre-fed with SG-deficient diet (-SG) or control diet for 2 weeks were intraperitoneally injected with LPS (25 mg/kg). ELISA detection of serum serine **(B)**, and cytokines including IL-1β, IL-6 and TNF-α **(C)** in the indicated groups.

**(D)**Representative images of hematoxylin and eosin (H&E) staining of lungs sections from the mice in the indicated groups are shown. Scale bars, 100 μm.

**(E)** qPCR analysis of inflammation-related cytokines and chemokines in lungs.

Data are presented as mean ± SEM. n = 3-4 per group (A-C and E); *p < 0.05, **p < 0.01, ***p < 0.001, two-tailed Student’s t-test.

**Figure S4**


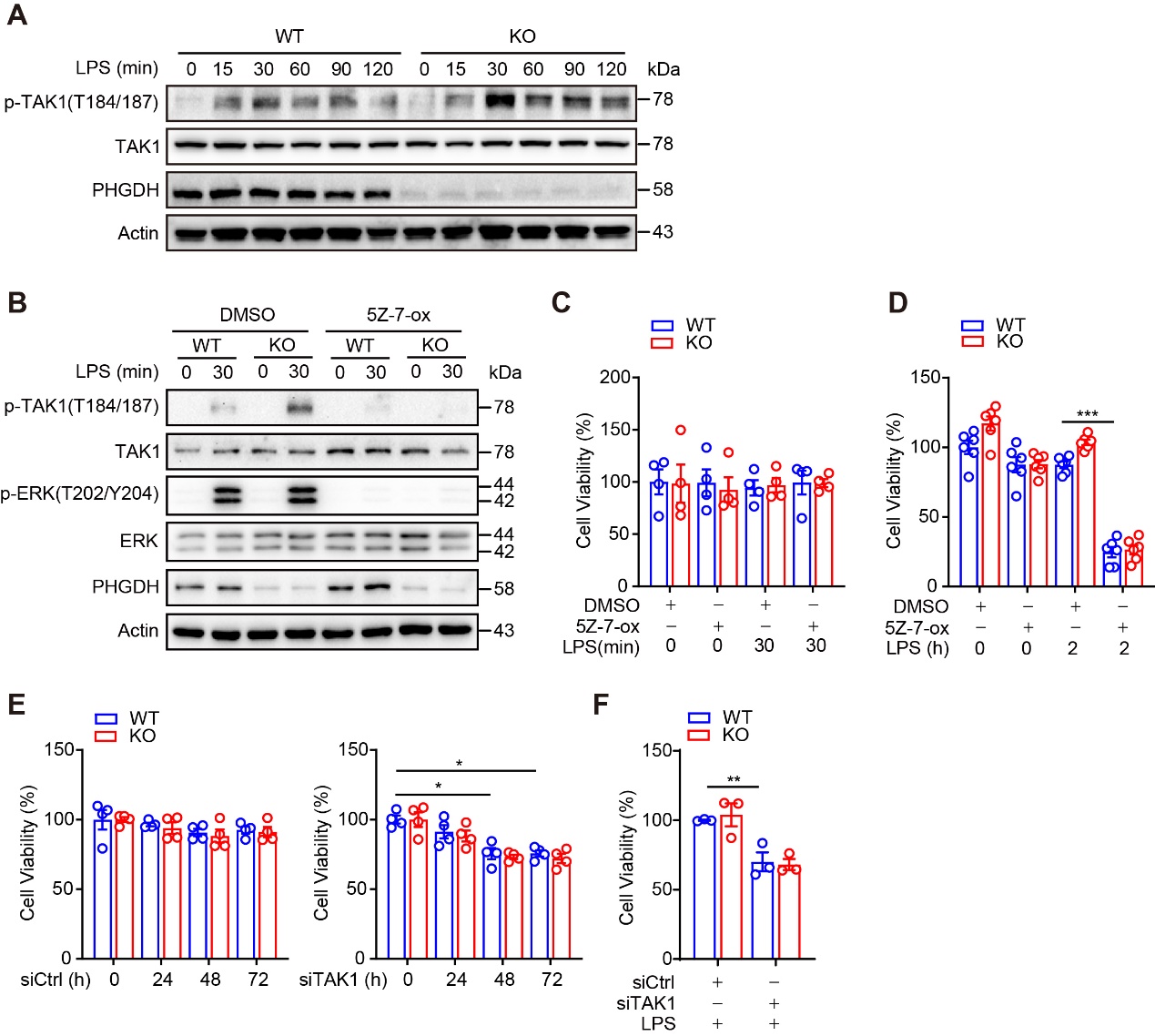


**Fig. S4** PHGDH suppresses LPS-mediated TAK1-NF-κB /MAPK signaling pathway

**A-B** Western blot analysis with the indicated antibodies. PHGDH-WT and PHGDH-KO BMDMs were pretreated for 2 h with or without the TAK1 inhibitor 5Z-7-ox at 100 nM, then treated with 500 ng/mL LPS for the indicated times.

**C-D** Cell viability was evaluated using CCK-8 assays in PHGDH-WT and PHGDH-KO BMDMs that were pretreated with 5Z-7-ox for 2 h and subsequently exposed to LPS for 30 min or 2 h.

**E****-F** Cell viability was evaluated using CCK-8 assays. PHGDH-WT and PHGDH-KO BMDMs were transfected with either siScramble (Scr) or siTAK1 for the indicated times (**E**) or for 48 h, followed by stimulation with LPS for 6 h (**F**).

Data are presented as mean ± SEM. n = 3-6 per group (C-F); *p < 0.05, **p < 0.01, ***p < 0.001, one-way ANOVA.

**Figure S5**


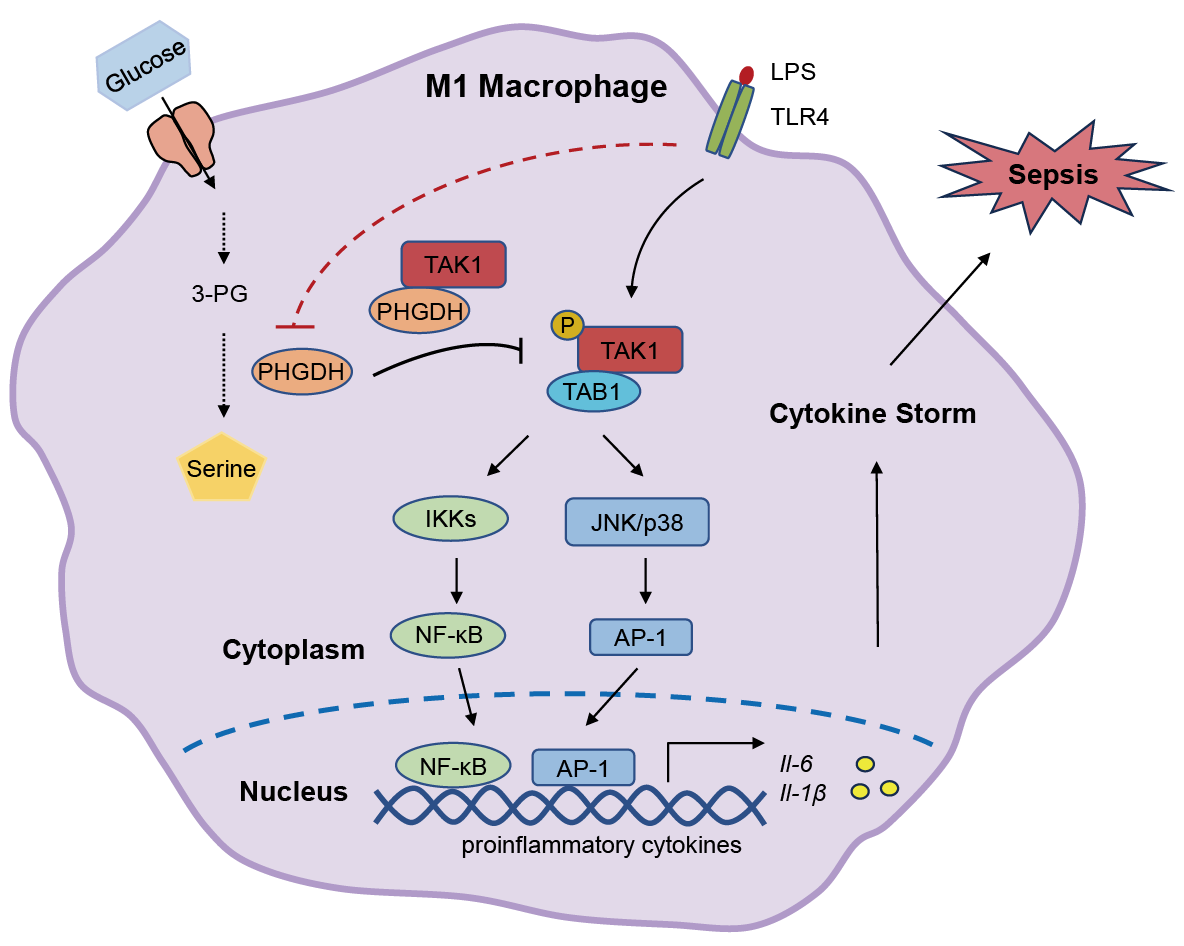


**Fig. S5** Phosphoglycerate dehydrogenase interacts with and inhibits TAK1 activity to mitigate septic shock

Model: Upon LPS stimulation or Gram-negative bacterial infection, expression of PHGDH is decreased. PHGDH inhibits the interaction between TAB1 and TAK1 by directly binding to TAK1, a non-metabolic function that suppresses TAK1 phosphorylation and downstream NF-κB and MAPK signaling pathways to reduce the production of anti-inflammatory cytokine IL-6 or IL-1β. In summary, our findings position macrophage PHGDH as a potential therapeutic target for septic shock.
